# Expanding the genetics and phenotypes of ocular congenital cranial dysinnervation disorders

**DOI:** 10.1101/2024.03.22.24304594

**Published:** 2024-03-26

**Authors:** Julie A. Jurgens, Brenda J. Barry, Wai-Man Chan, Sarah MacKinnon, Mary C. Whitman, Paola M. Matos Ruiz, Brandon M. Pratt, Eleina M. England, Lynn Pais, Gabrielle Lemire, Emily Groopman, Carmen Glaze, Kathryn A. Russell, Moriel Singer-Berk, Silvio Alessandro Di Gioia, Arthur S. Lee, Caroline Andrews, Sherin Shaaban, Megan M. Wirth, Sarah Bekele, Melissa Toffoloni, Victoria R. Bradford, Emma E. Foster, Lindsay Berube, Cristina Rivera-Quiles, Fiona M. Mensching, Alba Sanchis-Juan, Jack M. Fu, Isaac Wong, Xuefang Zhao, Michael W. Wilson, Ben Weisburd, Monkol Lek, Ocular CCDD Phenotyping Consortium, Harrison Brand, Michael E. Talkowski, Daniel G. MacArthur, Anne O’Donnell-Luria, Caroline D. Robson, David G. Hunter, Elizabeth C. Engle

**Affiliations:** 1 F.M. Kirby Neurobiology Center, Boston Children’s Hospital, Boston, MA, USA.; 2 Department of Neurology, Boston Children’s Hospital, Boston, MA, USA.; 3 Department of Neurology, Harvard Medical School, Boston, MA, USA.; 4 Broad Institute of MIT and Harvard, Cambridge, MA, USA.; 5 Howard Hughes Medical Institute, Chevy Chase, MD, USA.; 6 Department of Ophthalmology, Boston Children’s Hospital, Boston, MA, USA.; 7 Department of Ophthalmology, Harvard Medical School, Boston, MA, USA.; 8 Program in Medical and Population Genetics, Broad Institute of MIT and Harvard, Cambridge, MA, USA.; 9 Division of Genetics and Genomics, Boston Children’s Hospital, Harvard Medical School, Boston, MA, USA.; 10 Regeneron Pharmaceuticals, Tarrytown, NY, 10591, USA.; 11 Department of Pathology, University of Utah School of Medicine, Salt Lake City, UT, USA.; 12 Center for Genomic Medicine, Massachusetts General Hospital, Boston, MA, USA.; 13 Pediatric Surgical Research Laboratories, Massachusetts General Hospital, Boston, MA, USA.; 14 Division of Neuroradiology, Department of Radiology, Boston Children’s Hospital, Boston, MA, USA.; 15 Department of Radiology, Harvard Medical School, Boston, MA, USA.

## Abstract

**Purpose:**

To identify genetic etiologies and genotype/phenotype associations for unsolved ocular congenital cranial dysinnervation disorders (oCCDDs).

**Methods:**

We coupled phenotyping with exome or genome sequencing of 467 pedigrees with genetically unsolved oCCDDs, integrating analyses of pedigrees, human and animal model phenotypes, and *de novo* variants to identify rare candidate single nucleotide variants, insertion/deletions, and structural variants disrupting protein-coding regions. Prioritized variants were classified for pathogenicity and evaluated for genotype/phenotype correlations.

**Results:**

Analyses elucidated phenotypic subgroups, identified pathogenic/likely pathogenic variant(s) in 43/467 probands (9.2%), and prioritized variants of uncertain significance in 70/467 additional probands (15.0%). These included known and novel variants in established oCCDD genes, genes associated with syndromes that sometimes include oCCDDs (e.g., *MYH10, KIF21B, TGFBR2, TUBB6),* genes that fit the syndromic component of the phenotype but had no prior oCCDD association (e.g., *CDK13, TGFB2*), genes with no reported association with oCCDDs or the syndromic phenotypes (e.g., *TUBA4A, KIF5C, CTNNA1, KLB, FGF21*), and genes associated with oCCDD phenocopies that had resulted in misdiagnoses.

**Conclusion:**

This study suggests that unsolved oCCDDs are clinically and genetically heterogeneous disorders often overlapping other Mendelian conditions and nominates many candidates for future replication and functional studies.

## INTRODUCTION

Ocular congenital cranial dysinnervation disorders (oCCDDs) are rare neurogenic disorders that present as limited extraocular movement in one or multiple directions of gaze and/or ptosis. Extraocular muscles move the eyes and eyelids and are innervated by three cranial nerves (CN) that originate from brainstem motor nuclei: the oculomotor (CN3), trochlear (CN4), and abducens (CN6). oCCDDs result from defects in the development of these ocular motor neurons and/or their axons (Supplementary Fig. 1) and can occur in isolation or together with syndromic phenotypes.^1^ CN3-oCCDDs include congenital fibrosis of the extraocular muscles (CFEOM), congenital ptosis (ptosis), and Marcus Gunn jaw-winking syndrome (MGJWS). CFEOM is defined by non-progressive upgaze limitation, typically with ptosis and variable limitation of downgaze and horizontal gaze. Ptosis can also occur in isolation, or together with eyelid opening in response to jaw movement in MGJWS (MGJWS(+)ptosis).^2^ Rarely, MGJWS occurs without ptosis (MGJWS(−)ptosis), resulting in eye-opening with jaw movement. Inverse MGJWS (INV-MGJWS(−)ptosis) refers to eyelid closure upon jaw movement. CN4-oCCDDs include fourth nerve palsy (CN4-palsy), characterized by the inability to adduct and depress the eye, and Brown syndrome, which presents as limited elevation in adduction and can alternatively result from non-CN4-related mechanical restriction. CN6-oCCDDs include Duane retraction syndrome (DRS), congenital sixth-nerve palsy (CN6-palsy), and horizontal gaze palsy. DRS is characterized by limited ocular abduction and narrowing of the palpebral fissure with globe retraction on attempted adduction. CN6-palsy presents as limited abduction, and horizontal gaze palsy as limited abduction and adduction, both in the absence of globe retraction. Atypical oCCDDs that do not fit cleanly into these categories are referred to as “CCDD-not otherwise specified’’ (CCDD-NOS).

**Figure 1.**
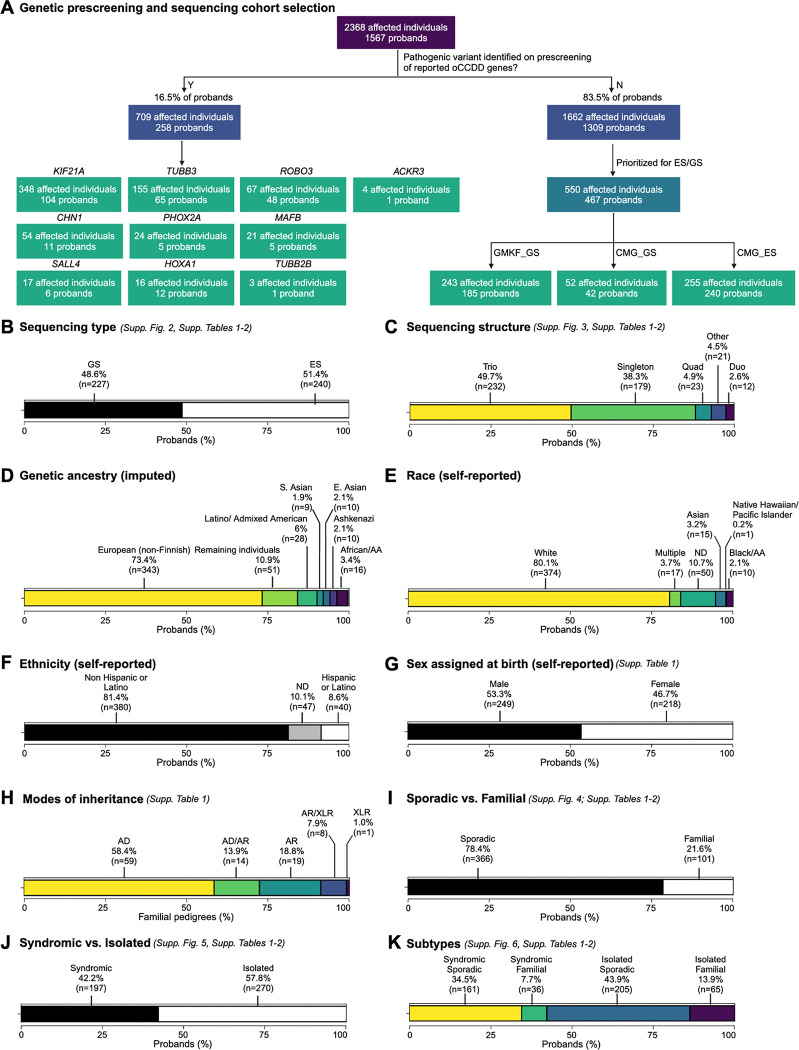
Selection and composition of sequenced probands in the ocular CCDD cohort. **A:** A large initial cohort of individuals with ocular CCDDs (oCCDDs) and their relatives were enrolled in our research study at Boston Children’s Hospital from August 1992 to June 2019. Individuals who had pathogenic variants in reported oCCDD genes identified on pre-screening were not included in the study (left side). The remaining individuals (right side) were prioritized for exome/genome sequencing if they screened negative for known common genetic etiologies of oCCDDs, had sufficient DNA quality and quantity, consented to broad genomic data sharing, and had additional syndromic features. Sequencing was performed through GMKF (GS) and the Broad CMG (GS and ES). **B-K:** Categorization of the proportions of 467 probands in the sequenced oCCDD cohort according to various metrics, as follows: B: Sequenced by GS versus ES. C: Sequenced as singletons, duos, trios, quads, or other (>4 total members of the pedigree sequenced). D: Imputed genetic ancestry groups. E: Self-reported race. F: Self-reported ethnicity. G: Self-reported sex assigned at birth. H: Modes of inheritance. I: Sporadic vs. familial. J: Syndromic vs. isolated. K: Both syndromic and sporadic, syndromic and familial, isolated and sporadic, or isolated and familial. For all relevant panels, accompanying supplementary figures and tables are denoted. Abbreviations: AA=African American, AD=autosomal dominant, AR=autosomal recessive, CCDD=congenital cranial dysinnervation disorder, CMG=Centers for Mendelian Genomics, E.=East, ES=exome sequencing, GMKF=Gabriella Miller Kids First, GS=genome sequencing, ND=not described, oCCDD=ocular congenital cranial dysinnervation disorder, S.=South, XLR=X-linked recessive.

Multiple oCCDD genes have been reported (Fig. 1A),as have genes underlying conditions that, rarely, are misdiagnosed as oCCDDs, including myasthenias, myopathies, or extraocular muscle maldevelopment syndromes.^3,4,5,6^ Most oCCDD genes were identified through analysis of large pedigrees and/or homogenous endophenotypes with shared etiologies. This leaves a large, genetically unsolved cohort of predominantly small, phenotypically heterogeneous pedigrees. To identify genetic causes and genotype/phenotype associations in this cohort, we performed exome or genome sequencing and prioritized single nucleotide variants (SNVs), small insertions/deletions (indels), and structural variants (SVs) to detect known and novel candidate protein-coding oCCDD etiologies.

## MATERIALS AND METHODS

The following sections are expanded upon in Supplementary Methods.

### Cohort enrollment, data collection, and phenotyping

We studied 467 genetically unresolved probands encompassing 11 oCCDDs (CFEOM, ptosis, MGJWS(+)ptosis, MGJWS(−)ptosis, INV-MGJWS(−)ptosis, CN4-palsy, Brown syndrome, DRS, CN6-palsy, horizontal gaze palsy, or CCDD-NOS) and their relatives (Supplementary Methods). Most probands (403/467, 86.3%) were pre-screened for pathogenic variants in reported oCCDD genes (Fig. 1A).

Demographics were collected via survey and self-reported by participants or their parents/legal guardians. Phenotypic data were obtained through retrospective review of clinical records, questionnaires, and updates from participants and their clinicians. Ocular motility data were reviewed by pediatric ophthalmologists, orthoptists, and neurologists (authors DGH, MCW, SM, ECE). Affected individuals were assessed for non-oculomotor syndromic phenotypes in 20 categories (Supplementary Methods). Brain magnetic resonance images (MRIs) were reviewed by a pediatric neuroradiologist (author CR) for image quality and for abnormalities of cranial nerves, extraocular muscles, structural brain, and other non-brain structures.

Individuals with an oCCDD and at least one major or two minor congenital anomalies were categorized as syndromic, while participants not meeting those criteria were categorized as isolated. Probands with or without a known family history of oCCDDs were designated as familial or sporadic. Co-occurring defects analysis determined whether oCCDDs and syndromic phenotypes co-occurred more frequently than by chance.^7^

### DNA sequencing

Exome/genome sequencing and genetic ancestry imputation were performed on 467 genetically unsolved pedigrees (550 affected and 1108 total individuals).

### SNV/indel analysis and prioritization

SNVs/indels were filtered using inheritance models, allele frequency, quality control, and variant annotation. Trios and larger pedigrees were assessed for frameshifting and non-frameshifting indels and for missense, nonsense, or splice site-altering SNVs in protein-coding genes. Singletons and duos were assessed only for variants in known oCCDD genes, strong candidate genes, genes mutated in >1 proband, and variants annotated as pathogenic/ likely pathogenic in ClinVar^8^ in additional genes. SNV/indels were prioritized using analyses of known biology and genotype/phenotype associations, animal models,^9^ statistical and pathway analyses of *de novo* variants (DNVs),^10,11^ and AlphaMissense scores^12^.

### SV analysis and prioritization

SVs perturbing coding sequences were identified, jointly genotyped, and annotated using GATK-SV (https://github.com/broadinstitute/gatk-sv) or GATK-gCNV and filtered for allele frequency, inheritance models, quality control, and variant annotation.^13,14^

### Interpretation of variant pathogenicity and submission to ClinVar

Using recommendations from the American College of Genetics and Genomics and Association for Molecular Pathology (ACMG/AMP)^15^ and the Clinical Genome Resource (ClinGen), SNVs/indels and SVs were prioritized and classified as pathogenic (P), likely pathogenic (LP), or uncertain significance (VUS), and submitted to ClinVar (Data Availability).^16^ Classified variants were grouped into five categories (Supplementary Methods).

### Genotype/phenotype correlations

For pedigrees with highlighted genetic findings, clinical features were reported using Human Phenotype Ontology (HPO)^17^ and assessed for genotype/phenotype associations.

## RESULTS

### Definition of the oCCDD cohort

At the onset of this project, we had enrolled 1567 pedigrees with oCCDDs; the phenotype was familial in 364 (23.4%). A genetic etiology had been identified for 258 pedigrees (16.5%), which were not included in this study (Fig. 1A, Supplementary Results).

Among 1309 unsolved pedigrees, 467 were sequenced (550 affected and 1108 total individuals). Pedigrees were eligible for exome/genome sequencing if they screened negative for common genetic etiologies, had sufficient DNA quality and quantity, and consented to data sharing in controlled access repositories. Pedigrees with familial oCCDDs and/or syndromic features were further prioritized. 227 (48.6%) and 240 (51.4%) pedigrees had genome and exome sequencing, respectively. Most were sequenced as trios (49.7%) or singletons (38.3%), with fewer duos (2.6%), quads (4.9%), or “other” pedigrees (>4 individuals; 4.5%). Five pedigrees for which we reported a genetic etiology during the study were included (Pedigrees 38, 48, 56, 98, 144).^18–22^ Clinical genetic testing before exome/genome analysis explained the syndromic phenotypes of two unrelated individuals (proband ENG_AKL and affected individual 178_04). Genetic imputation revealed European ancestry in 73.4% of probands; this was corroborated by self-reported race and ethnicity (reported at a cohort-wide level). Male-assigned sex at birth and consanguinity were reported in 53.0% and 4.3% of probands, respectively. The oCCDD was familial in 101 pedigrees (21.6%), of which 58.4% displayed autosomal dominant inheritance. 270/467 probands (57.8%) had an isolated oCCDD, and almost half had both isolated and sporadic oCCDDs (43.9%). See Fig. 1A–K, Supplementary Results, Supplementary Figs. 2–6, Supplementary Tables 1–2.

**Figure 2.**
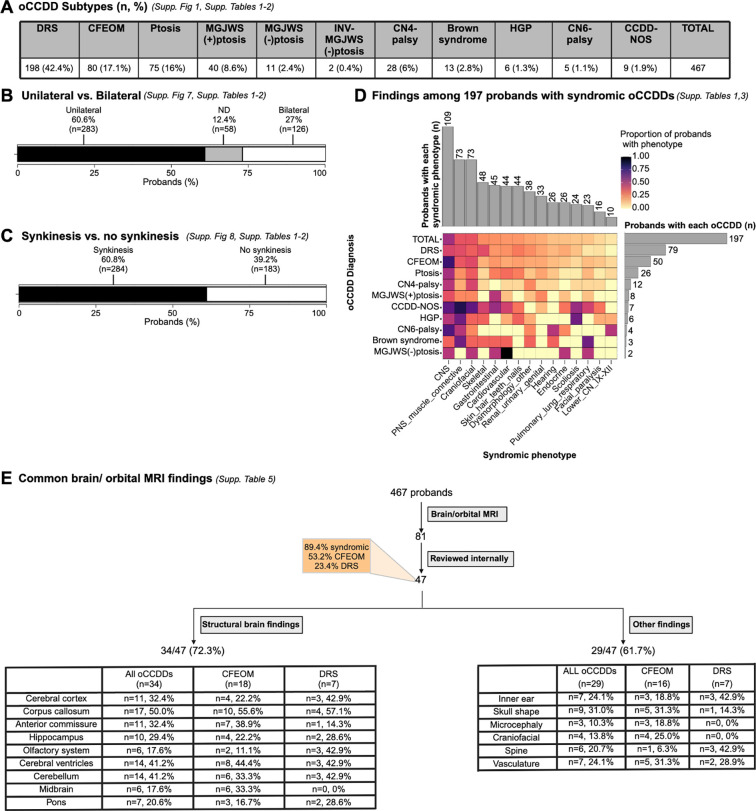
Phenotypes among sequenced probands in the ocular CCDD cohort. **A:** Numbers and percentages of probands with each oCCDD subtype. **B:** oCCDD laterality among all oCCDD probands. **C:** Evaluation of synkinesis among all oCCDD probands. **D:** Syndromic findings among the 197 probands with syndromic oCCDDs. Proportions of probands with each syndromic finding are represented colorimetrically within the heatmap. Gray bars on the right side of the heatmap show the total number of syndromic probands with each oCCDD subtype (i.e. 79 probands had DRS), while gray bars above the heatmap show the total number of probands with involvement in the corresponding syndromic category beneath the heatmap (e.g., 109 total probands had CNS involvement). **E:** Brain/orbital MRI findings among oCCDD probands. Of the 467 sequenced oCCDD probands, 81 had clinically obtained MRIs, 47 of which were available for review (of whom 89.4% had a syndromic oCCDD). Proportions of individuals with various structural brain anomalies and other findings are provided. For all relevant panels, accompanying supplementary figures and tables are denoted. Abbreviations: CCDD=congenital cranial dysinnervation disorder, CCDD-NOS=CCDD not otherwise specified, CFEOM=congenital fibrosis of the extraocular muscles, CMG=Centers for Mendelian Genomics, CN4-palsy=fourth nerve palsy, CN6-palsy=sixth nerve palsy, CNS=central nervous system, DRS=Duane retraction syndrome, E.=East, ES=exome sequencing, GMKF=Gabriella Miller Kids First, GS=genome sequencing, HGP=horizontal gaze palsy, INV-MGJWS(−)ptosis=inverse Marcus Gunn jaw-winking synkinesis without congenital ptosis, MGJWS(+)ptosis=Marcus Gunn jaw-winking synkinesis with congenital ptosis, MGJWS(−)ptosis=Marcus Gunn jaw-winking synkinesis without congenital ptosis, ND=not described, PNS=peripheral nervous system, Ptosis=congenital ptosis, S.=South, XLR=X-linked recessive.

DRS was the most common oCCDD in the cohort (n=198, 42.4%). oCCDDs were unilateral (60.6%), bilateral (27.0%), or of unknown laterality (12.4%), and synkinesis was described in 60.8%. Syndromic findings were noted in 197 probands (42.2%). We identified co-occurring phenotypes underlying known syndromes but no distinguishable novel syndromes. See Fig. 2A–D, Supplementary Results, Supplementary Figures 7–8, Supplementary Tables 1–4.

### Brain and Orbital MRI findings

Brain/orbital MRIs for 47 probands were available for review. Of these probands, 53.2% had CFEOM, 23.4% had DRS, and 89.4% were syndromic. Thirteen scans were optimized for cranial nerve/extraocular muscle detection, and all had ocular cranial nerve and/or extraocular muscle abnormalities. In most, the affected cranial nerves/extraocular muscles on MRI were consistent with the clinically diagnosed oCCDD, though occasional images revealed phenocopies such as extraocular muscle-tethering orbital bands (Fig. 2E, Supplementary Results, Supplementary Table 5).

Scans revealed structural brain and non-brain anomalies in 34/47 (72.3%) and 29/47 (61.7%) of probands, respectively. While some have been reported with oCCDDs (e.g., inner ear findings),^3^ others may have been previously underrecognized (e.g., vascular anomalies; Fig. 2E, Supplementary Results, Supplementary Table 5).

### SNV/indel analyses

**Figure 3.**
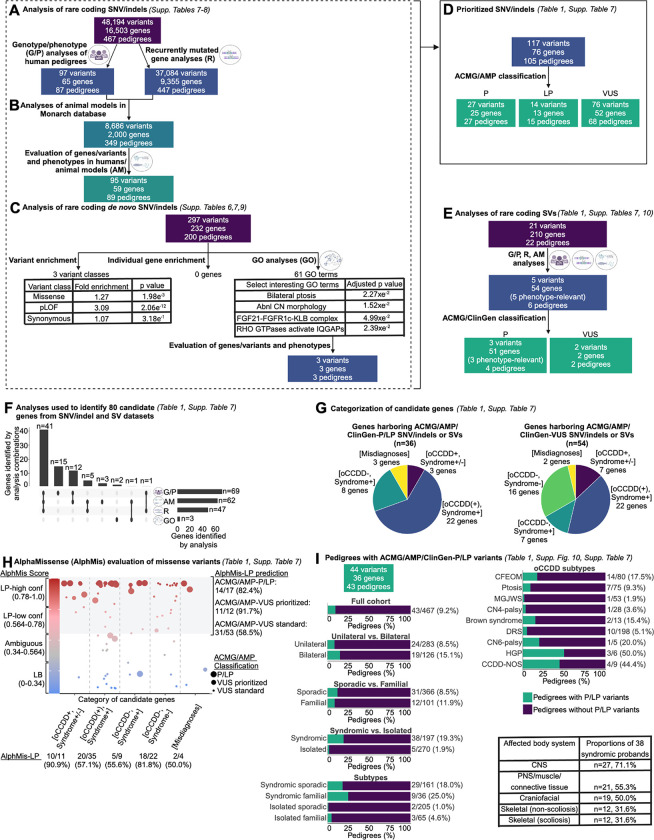
Workflow of genetic analyses. **A-C:** Workflow for analyses of rare coding SNVs and indels. A: Pedigree-based genotype/phenotypes (G/P) analyses and recurrently mutated gene analyses. Rare coding SNVs/indels in all 467 pedigrees were identified and subjected to genotype/phenotype analyses as described (Supplementary Methods, “SNV/indel filtering and prioritization” and “Biological prioritization of SNVs/indels” sections). Genes that had SNVs/indels meeting the parameters defined in Supplementary Methods, “SNV/indel filtering and prioritization” and that were mutated in more than one pedigree were defined as recurrently mutated genes. Recurrently mutated genes were queried in all 467 pedigrees regardless of specific oCCDD diagnosis. **B:** Workflow for animal model analyses (“AM” analyses). Each of the 65 genes that harbored rare coding SNVs/indels derived from G/P analyses and the 9,355 genes identified from recurrently mutated gene (“R”) analyses (from ‘A’) were annotated for putatively relevant animal model phenotypes in the Monarch database, which yielded 2,000 genes with putatively oCCDD-relevant animal models. 59 candidate genes / 95 variants were prioritized from this analysis (defined in Supplementary Methods, “Animal model analyses” section). **C:** Workflow for statistical and gene ontology (GO) analyses of *de novo* variants (DNVs). Rare coding DNV SNV/indels were assessed for overall enrichment of various classes of DNVs (left) and enrichment of DNVs in individual genes (middle) through DenovolyzeR, and for enrichment of genes in specific pathways through GO analysis (right). Numbers of total and specific classes of enriched GO terms are displayed, along with select enriched GO terms of biological interest. Genes derived from the GO terms “FGF21-FGFR1c-KLB complex” and “RHO GTPases activate IQGAPs” were nominated for ACMG/AMP classification. **D:** ACMG/AMP classification. In total from the G/P, R, AM, and/or GO analyses of SNVs/indels in (A-C), 117 variants in 76 genes were prioritized and classified by the ACMG/AMP criteria. The gene/variant counts were derived from (A-C) minus redundant genes/variants. In total, only 76 distinct genes are represented among the three ACMG/AMP classification categories, as some genes had variants in more than one classification category. **E:** Rare coding SVs were prioritized using G/P, R, and AM analyses as described for SNV/indels, which led to the nomination of 5 SVs for classification by ACMG/ClinGen criteria. **F:** UpSet plot summarizing the combinations of analyses (G/P, AM, R, and GO) used to derive the 80 candidate genes whose variants were nominated for ACMG/AMP/ClinGen classification of SNV/indels (76 genes from D) or SVs (4 genes not overlapping with SNV/indels). Vertical bars denote numbers of candidate genes identified by each combination of analyses. Horizontal bars denote numbers of genes identified by each analysis type in total (numbers were obtained by adding genes with prioritized SNV/indels plus genes with prioritized SVs minus genes represented redundantly between the SNV/indel and SV classes; Supplementary Table 7). **G:** Classification of candidate genes harboring ACMG/AMP/ClinGen-P/LP SNV/indels and SVs (left chart), or ACMG/AMP/ClinGen-VUS SNV/indels and SVs (right chart). Among the 80 genes with P/LP or VUS variants (F), 10 were represented in both categories. Genes were stratified into five categories. Purple [oCCDD+,Syndrome+/−]: genes that were definitively associated with oCCDDs before this study and were genetically pre-screened in most probands. Blue [oCCDD(+),Syndrome+]: genes that had at least occasional prior oCCDD association but were typically part of specific monogenic syndromes and thus not pre-screened. Dark green [oCCDD−,Syndrome+]: genes that fit the syndromic component of each proband’s phenotype but that, to our knowledge, have no prior oCCDD association. Light green [oCCDD−,Syndrome−]: genes that, to our knowledge, had no reported association with either the oCCDD or non-CCDD phenotype of the probands who harbor them. Yellow [Misdiagnoses]: genes associated with alternative non-neurogenic/ non-CCDD etiologies and represent misdiagnoses or oCCDD phenocopies. **H:** AlphaMissense (AlphMis) could be used to assess 82 of the ACMG/AMP classified missense SNVs. X-axis: Numbers of missense variants and percent scored as AlphMis-LP in each of the five categories as defined in (G). Y-axis (left side): AlphMis scores on a scale of zero to one accompanied by the corresponding pathogenicity score (LB, Ambiguous, LP-low confidence, LP-high confidence). Scores are color-coded from blue (LB) to red (LP). Each dot on the plot represents a separate missense variant, and dot sizes correspond with ACMG/AMP classifications (large dots: ACMG/AMP-P/LP variants, medium dots: strongly prioritized ACMG/AMP-VUS, small dots: standardly prioritized ACMG/AMP-VUS). Strongly prioritized ACMG/AMP-VUS are the missense VUS denoted in Table 1; while these are formally classified as ACMG/AMP-VUS, we concluded that these variants have compelling biological and/or genotype/phenotype evidence and are most likely to be substantiated over time. Standardly prioritized ACMG/AMP-VUS are all additional missense ACMG/AMP-VUS denoted in Supplementary Table 7 that we prioritized but that currently have less supportive evidence than the strongly prioritized VUS. High-confidence and low-confidence AlphMis-LP variants are encompassed by the gray shaded region of the graph and compared to their independently obtained ACMG/AMP classifications (right side of the graph); numerical summaries are provided for each, for instance: 14/17 (82.4%) ACMG/AMP-P/LP variants were also scored as LP by AlphaMissense. **I:** Rates of ACMG/AMP/ClinGen-P/LP variants (SNVs, indels, and SVs) obtained for the full cohort (left) and individual subgroups (top right). Rates are given as the number of pedigrees within each group who had ACMG/AMP/ClinGen-P/LP variant(s) relative to the total number of pedigrees within that group. Green= pedigrees with ACMG/AMP/ClinGen-P/LP variant(s); Purple= pedigrees without ACMG/AMP/ClinGen-P/LP variant(s). Among 38 syndromic probands who had ACMG/AMP/ClinGen-P/LP variant(s), the most frequently affected body systems are shown (bottom right). For all relevant panels, accompanying supplementary figures and tables are denoted. Abbreviations: AM=animal model analyses, abnl=abnormal, ACMG=American College of Genetics and Genomics, AlphMis=AlphaMissense, AMP=Association for Molecular Pathology, CCDD=congenital cranial dysinnervation disorder, CCDD-NOS=CCDD not otherwise specified, CFEOM=congenital fibrosis of the extraocular muscles, ClinGen=Clinical Genome Resource, CN=cranial nerve, CN4-palsy=fourth nerve palsy, CN6-palsy=congenital sixth nerve palsy, CNS=central nervous system, conf=confidence, DRS=Duane retraction syndrome, GO=gene ontology analyses, G/P=genotype/phenotype analyses, HGP=horizontal gaze palsy, HPO=human phenotype ontology, indel=small insertion/deletion, LB=likely benign, LP=likely pathogenic, MGJWS=Marcus Gunn jaw-winking synkinesis, misc=miscellaneous, oCCDD=ocular congenital cranial dysinnervation disorder, P=pathogenic, pLOF=predicted loss of function (nonsense, splicing, or frameshift), PNS=peripheral nervous system, ptosis=congenital ptosis, R=recurrently mutated gene analyses, SNV=single nucleotide variant, SV=structural variant, VUS=variant of uncertain significance.

Among all 467 pedigrees, SNV/indel filtering yielded 48,194 rare coding variants in 16,503 genes (Fig. 3A). Among 276 pedigrees with ≥3 individuals sequenced, the yield was 6,102 variants in 4,288 genes (Supplementary Table 6). To further prioritize these, we implemented analyses of genotype/phenotypes, animal models, and statistical and pathway analyses of DNVs.

We first analyzed individual pedigrees and performed genotype/phenotype correlations, prioritizing 97 variants in 65 genes among 87 pedigrees. We identified strong candidate variants among 13 pedigrees in 6 oCCDD genes (*KIF21A*, *TUBB3*, *PHOX2A, CHN1*, *MAFB*, *ROBO3*). Next, we prioritized additional genes/variants based on predictive scores, functional annotations, and reported associations with the oCCDD and/or syndromic phenotype in the corresponding pedigree (Table 1, Fig. 3A, Supplementary Results, Supplementary Table 7).

We identified strong candidate variants in *TUBA1A, TUBB6, TUBB4A,* and *TUBB*, complementing our previous reports of tubulin-encoding *TUBB3* and *TUBB2B* as oCCDD genes.^1^ We reported *TUBA1A* as a novel CFEOM gene during this study (pedigree 38)^21^ and subsequently identified an additional variant in syndromic sporadic DRS pedigree 170. We identified a *TUBB6* p.(Glu410Lys) variant in syndromic familial ptosis pedigree ENG_CML; this substitution has been reported as P/LP for tubulinopathies in four paralogs (*TUBB2A, TUBB2B, TUBB3,* and *TUBB4A;* ClinVar Variation IDs: 986830, 1195195, 6967, 135658). Moreover, a separate *TUBB6* missense variant was reported in a 5-generation syndromic ptosis pedigree (MIM617732).^23^ Similarly, we identified a *TUBA4A* heterozygous p.(Arg390His) variant in isolated sporadic ptosis pedigree ENG_IM; this residue aligns with tubulinopathy-associated residues in four paralogs (*TUBA1A, TUBB2A, TUBB2B,* and *TUBB3;* ClinVar Variation IDs: 488628, 423490, 418531, 450183, 1214258, 160177, 1203166, 429413, 1320230, 219257). These findings support the putative pathogenicity of these missense changes. Separate missense or loss-of-function variants in *TUBA4A* have been reported in amyotrophic lateral sclerosis (MIM616208) but not with oCCDDs. Finally, we identified strong candidate variants in additional tubulin-encoding genes *TUBB4A* in isolated familial Brown syndrome and *TUBB* in isolated sporadic CFEOM (pedigrees 216, ENG_0678; Supplementary Table 7).

We identified heterozygous *MYH10* variants in five pedigrees with CN3-oCCDDs: ENG_CKM (isolated familial CFEOM), ENG_ASW (syndromic sporadic CFEOM), ENG_YY (isolated sporadic MGJWS(+)ptosis), ENG_PJ (isolated sporadic MGJWS(+)ptosis), and ENG_CGO (isolated sporadic MGJWS(−)ptosis) (Supplementary Table 7, Supplementary Figure 9). *MYH10* is highly missense-constrained within humans (missense z=5.006)^24^ and encodes a nonmuscle myosin that modulates actin dynamics. Separate *MYH10* heterozygous missense or loss-of-function variants were recently associated with neurodevelopmental phenotypes, which included ptosis in 3 individuals, lateral rectus muscle weakness in 1 individual, and CN5/CN7 palsy in 1 individual.^25,26^ Additional *MYH10* missense variants have been reported in probands with ptosis, coloboma, and craniofacial dysmorphisms (Scheidecker et al., personal communication).

Among 8 probands, we identified 12 variants in 7 genes associated with ciliopathies including Joubert syndrome (*KIAA0586, ARMC4, BBS1, CEP83, ARMC9, TOGARAM1, WDR5;* Supplementary Table 7). In Joubert syndrome, eye movement abnormalities and ptosis are common, and oCCDD-like phenotypes are reported infrequently.^27^ Our findings substantiate the association between genetically diverse ciliopathies and oCCDDs.

Finally, 4 probands harbored a variant in one of three TGF-beta pathway genes (*FBN1, TGFB2, TGFBR2*; Supplementary Table 7). These genes are associated with connective tissue disorders that are occasionally accompanied by ocular abnormalities including putative CFEOM,^28^ but these are not strongly associated oCCDD genes.

Among 447 pedigrees, we identified 9,355 recurrently mutated genes. These were prioritized if also nominated by additional analyses of genotype/phenotypes above, or of animal models or DNVs below.

For many oCCDD genes, animal models have recapitulated human oCCDD phenotypes.^1,3,4^ Thus, we queried whether strong candidate genes identified from genotype/phenotype analysis or recurrently mutated candidate genes were annotated for oCCDD-relevant animal model phenotypes in the Monarch database^9^, and further prioritized 95 variants in 59 genes among 89 pedigrees (Fig. 3B, Supplementary Results, Supplementary Table 8). We identified a homozygous *ECEL1* variant that disrupts a residue involved in disulfide bonding in pedigree 223 with familial DRS and arthrogryposis. Biallelic *ECEL1* variants are reported to cause distal arthrogryposis (MIM615065)^29^ that, rarely, is accompanied by an oCCDD. Supporting *ECEL1* variants in DRS pathogenicity, *Ecel1* mouse models have abnormal CN6 innervation (Table 1; Supplementary Tables 7–8).^30^ Animal model analyses also prioritized candidate variants in genes without known human oCCDD involvement (*KIF5C*, *NES, CUX1, GNAS, FER, ACTR1B, OLIG2,* and *SEMA3F;* Supplementary Results; Supplementary Tables 7–8). Among these, the neuronal kinesin-encoding gene *KIF5C* was mutated in three singletons: ENG_1561 (syndromic sporadic DRS), ENG_ABE (syndromic familial DRS), and ENG_UV (isolated sporadic CCDD-NOS with intermittent blinking during smiling). *KIF5C* is highly missense-constrained within humans (missense z=4.054).^24^ Distinct *KIF5C* variants have been identified in human cortical brain malformations with variable syndromic involvement;^31^ unfortunately, our probands did not have MRIs to evaluate cortical brain malformations. Intriguingly, *Kif5c*^−*/*−^ mice have fewer CN6 motor neurons,^32^ consistent with DRS. Moreover, downregulation of the oCCDD gene *TUBB3* increases *KIF5C* motility and cargo transport.^33^ While this is an intriguing candidate, all pedigrees with *KIF5C* variants in our cohort are singletons, and the syndromic phenotypes in the two DRS probands are disparate from one another and from known *KIF5C*-associated syndromic findings.

We next identified and performed statistical and pathway analyses of 297 DNVs in 232 genes among 200 pedigrees (Fig. 3C). Probands had an enrichment of missense and predicted loss-of-function DNVs but not synonymous DNVs (fold enrichment=1.27, 3.09, 1.07; p=1.98e^−3^, 2.06e^−12^, 3.18e^−1^; Poisson test). Because DNVs were not significantly enriched in any individual gene (Supplementary Results), we tested enrichment among genes associated with shared HPO terms, signaling pathways, or protein complexes via GO analysis. Many HPO terms were enriched (Supplementary Table 9), including terms supporting the involvement of DNV-genes in oCCDDs, such as bilateral ptosis and abnormal cranial nerve morphology (p_adj_=2.27e^−2^ and 1.52e^−2^). Additionally, we identified pathways or protein complexes that highlighted novel candidate genes. For instance, the enriched pathway term “RHO GTPases activate IQGAPs” (p_adj_=2.39e^−2^) included oCCDD genes (*TUBB3, TUBA1A, ACTB*), but also highlighted *CTNNA1*, which had a DNV in pedigree 99 with syndromic sporadic CFEOM (Supplementary Tables 7,9). *CTNNA1* variants have been associated with retinopathies (MIM116805) but not with oCCDDs. The term for the FGF21-FGFR1c-KLB protein complex was also enriched (p_adj_=4.99e^−2^), with DNVs in *FGF21* in syndromic sporadic CFEOM pedigree 91 and in *KLB* in syndromic sporadic CCDD-NOS pedigree ENG_CKP (Supplementary Tables 7,9). Notably, variants in other FGF signaling genes are reported to cause phenotypes present in pedigree 91, including hypogonadism, syndactyly, craniofacial dysmorphisms, and developmental delay.^34^ Moreover, hypogonadism has been reported in *Fgf21*^−*/*−^ mice.^35^ Part of the syndromic phenotype in ENG_CKP is craniosynostosis, a phenotype associated with other FGF signaling genes but not yet *KLB*. Finally, a single *Klb*^−*/*−^ mouse image demonstrates periorbital abnormalities reminiscent of ptosis or globe retraction.^36^

### ACMG/AMP classification of prioritized SNVs/indels

Through qualitative manual review of genes obtained from analysis of genotype/phenotypes, animal models, and pathways above, we selected a subset of 117 SNV/indels among 105 probands for ACMG/AMP classification (Fig. 3D), including the four probands for whom we reported a causal SNV/indel.^18,19,21,22^ For this subset, we also annotated HPO terms and performed additional extensive genotype/phenotype correlations for oCCDD and syndromic phenotypes (Supplementary Tables 1,7).

In total, ACMG/AMP classification identified 41 P/LP SNVs/indels among 39/467 probands (8.4%; Fig. 3D, Table 1, Supplementary Table 7). Three probands had compound heterozygous variants in a single gene (pedigrees 71, 239, ENG_AGZ), and one proband had variants in 2 genes that contributed to a blended phenotype (pedigree 269; variants in *ARMC4* and *ARMC9*). Additionally, 68/467 probands (14.6%) harbored 76 prioritized VUS SNV/indels among 52 genes; the VUS was compound heterozygous with a P/LP variant in pedigrees 193 and ENG_AZW. In pedigree ENG_COX, only a single allele was identified in a gene for which biallelic variants typically cause the phenotype. Some VUS have more supportive evidence, suggesting higher likelihood of their being substantiated over time (Table 1).

### SV analyses and classification with ACMG/ClinGen criteria

In 22 pedigrees, exome/genome analyses detected 21 rare candidate SVs that were predicted to perturb protein-coding sequences (Fig. 3E, Supplementary Results, Supplementary Table 10). We prioritized 5 SVs for ACMG/ClinGen classification^16^; these encompassed gene(s) associated with phenotypes consistent with the probands’. Three deletions among 4 probands were classified as pathogenic: an *HDAC8* deletion in pedigree 13 with syndromic sporadic ptosis, whose phenotype was consistent with *HDAC8*-associated conditions^37^; a deletion including *GCH1* in syndromic familial ptosis pedigree ENG_BS, which explained their ptosis and DOPA-related dystonia^38^; and a chr10q26 deletion in syndromic sporadic DRS pedigrees 233 and 131, which we reported recently (https://doi.org/10.1101/2023.12.22.23300468). The remaining two deletions scored as ACMG/ClinGen-VUS; they likely explained the clinical phenotypes but did not meet pathogenic classification thresholds. These included a partial deletion of *MACF1* in syndromic sporadic CN6-palsy pedigree 98, which we reported during the course of this study,^20^ and a partial deletion of *SALL4* in syndromic sporadic DRS pedigree ENG_DQ, whose phenotype was consistent with *SALL4-*associated conditions.^1^ See Table 1, Supplementary Table 7.

### Categorization of genes with causal and candidate SNVs/indels or SVs

The genes that harbored causal and candidate SNVs/indels or SVs (classified as P/LP or VUS, respectively) were identified through various combinations of analyses and fell into five distinct categories: 1) [oCCDD+,Syndrome+/−]: genes that were definitively associated with oCCDDs before this study and were genetically pre-screened in most probands. 2) [oCCDD(+),Syndrome+]: genes that had at least occasional prior oCCDD association but were typically part of specific monogenic syndromes and thus not pre-screened. 3) [oCCDD−,Syndrome+]: genes that fit the syndromic component of each proband’s phenotype but had no prior oCCDD association. 4) [oCCDD−,Syndrome−]: genes that had no reported association with either the oCCDD or non-CCDD phenotype of the probands who harbor them. 5) [Misdiagnoses]: genes associated with alternative non-neurogenic/ non-CCDD etiologies and represent misdiagnoses or oCCDD phenocopies. Genes harboring P/LP or VUS were predominantly in the [oCCDD(+),Syndrome+] category (Fig. 3F–G, Supplementary Results, Supplementary Table 7).

### Evaluation of classified or recurrent missense SNVs with AlphaMissense

To complement our genetic analyses, which depended on known biology and oCCDD etiologies, we evaluated ACMG/AMP-classified or recurrent missense variants with AlphaMissense (Fig. 3H, Supplementary Table 7).^12^ AlphaMissense labels predicted deleterious variants as “likely pathogenic” (LP), with the caveat that these pathogenicity assertions are not corroborated using non-computational evidence as for ACMG/AMP classifications. AlphaMissense scores 22.8 of 71 million potential proteome-wide missense variants as LP (32.1%).^12^ Of 41 ACMG/AMP-P/LP variants, 17 were missense and AlphaMissense scored 14 as LP (82.4%). Of 76 ACMG/AMP-VUS, 65 were missense and AlphaMissense scored 42 as LP (64.6%). Interestingly, among these 65 VUS, we highlighted 12 with especially strong genotype/phenotype correlations (Table 1); AlphaMissense scored 11 of these as LP (91.7%). Of the remaining 53, AlphaMissense scored only 31 as LP (58.5%). Finally, of 823 recurrent heterozygous missense variants among all 467 probands, 743 could be evaluated by AlphaMissense. Of these, only 83 scored as LP (11.2%); this count included a recurrent ACMG/AMP-pathogenic *KIF21A* p.(Arg954Trp) variant. AlphaMissense scores support our prioritized variants as deleterious, and also suggest that recurrent heterozygous variants may not be a large genetic contributor.

We next evaluated missense variants within the 5 gene categories above with AlphaMissense (Fig. 3H). In [oCCDD+,Syndrome+/−] genes – which had known prior oCCDD associations – 10/11 variants were scored as LP by AlphaMissense (90.9%); only 3 were ACMG/AMP-P/LP, suggesting that the remaining VUS are strong candidates for future study. AlphaMissense scored 1/11 variants as likely benign: a *TUBB3* ACMG/AMP-VUS p.(Cys124Ser), which we had ACMG/AMP-classified because of *TUBB3*-CFEOM associations but we had felt was less compelling a priori. [oCCDD(+),Syndrome+] contained 20/35 AlphaMissense-LP variants (57.1%), 6 of which were also ACMG/AMP-P/LP. Interestingly, two [oCCDD(+),Syndrome+] variants in *PTPN11* and *TRPV4* were ACMG/AMP-pathogenic but scored as ambiguous by AlphaMissense. In [oCCDD−,Syndrome+], 5/9 variants (55.6%) were AlphaMissense-LP, 3 of which were also ACMG/AMP-pathogenic, and a variant in *SCN1A* was pathogenic by ACMG/AMP but likely benign by AlphaMissense. Interestingly, [oCCDD-,Syndrome-], encompassing genes with no reported association with either the oCCDD or non-CCDD phenotypes, included 18/22 AlphaMissense-LP variants (81.8%); all were ACMG/AMP-VUS. Finally, in [Misdiagnoses] were 2/4 AlphaMissense-LP variants (50.0%), both of which were ACMG/AMP-P/LP. Cumulatively, these results suggest that AlphaMissense may aid in prioritizing ACMG/AMP-VUS.

### Characteristics of oCCDD probands with ACMG/AMP or ACMG/ClinGen P/LP variants

Collectively, SNV/indel and SV analyses identified ACMG/AMP/ClinGen-P/LP variant(s) in 43/467 pedigrees (9.2%; Fig. 3I, Supplementary Results, Supplementary Fig. 10, Supplementary Table 7). These variants explained the phenotype fully in 13 pedigrees and partially in 30 (Table 1). In the partially explained pedigrees, putative phenotype expansions were identified for oCCDDs (n=13), syndromic phenotypes (n=3), or both oCCDDs and syndromic phenotypes (n=14). In two cases, the P/LP allele was compound heterozygous with a VUS. Fourteen highly prioritized VUS were identified in 13 genes among 14 pedigrees (Table 1). Moreover, we identified additional VUS in known and novel candidate genes which we believe merit future study (Supplementary Table 11).

P/LP variants were more common in probands with syndromic than isolated oCCDDs (38/197 versus 5/270; chi-square test of independence, Χ^2^=41.43, df=1, p=1.2e^−10^). The most common affected body systems were CNS (27/38, 71.1%), PNS/muscle/connective tissue (21/38, 55.3%), craniofacial (19/38, 50.0%), skeletal (non-scoliosis) (12/38, 31.6%), and skeletal (scoliosis) (12/38, 31.6%; Fig. 3I). Probands with P/LP variants had a mean of 3.67 affected body systems, and 25/38 syndromic probands (65.8%) had >2 body systems affected.

While P/LP variants were higher among familial than sporadic pedigrees (12/101, 11.9%; 31/366, 8.5%), most probands with P/LP variants had sporadic syndromic oCCDDs (29/43, 67.4%). Though bilateral oCCDDs were infrequent in our overall cohort, they were common among probands with P/LP variants (126/467 versus 19/43; chi-square test of independence, Χ^2^=5.73, df=1, p=0.017). Rates of P/LP variants varied among oCCDD subphenotypes (Fig. 3I).

## DISCUSSION

As the largest oCCDD cohort reported to date, our study contributes detailed clinical and MRI data to our public exome/genome datasets. Moreover, within the cohort we collate (1) manually curated genes that harbor variants and have putatively relevant animal models; (2) genes and pathways highlighted by GO analyses of DNVs; (3) prioritized pathogenicity-classified variants with comprehensive genotype/phenotype correlations; and (4) AlphaMissense scoring of highlighted missense variants. These resources should facilitate future oCCDD gene discovery.

Though most of our cohort was prescreened for variants in known oCCDD genes to enable novel gene discovery, 9.2% of our cohort had P/LP variants – many of which were not in our original list of oCCDD genes to pre-screen – and additional cases had strong VUS. Many pedigrees had diverse syndromic phenotypes and were sequenced to identify overlap between oCCDDs and other congenital defects; indeed, exomes/genomes identified diverse genes and pathways associated with many Mendelian conditions. These often had known roles in syndromic phenotypes but unknown or infrequent prior oCCDD connection ([oCCDD(+),Syndrome+], [oCCDD−,Syndrome+]). While some of these may explain only the syndromic phenotypes in the probands who harbor them, others likely represent true oCCDD phenotype expansions for known disorders. Examples included candidate variants in *MYH10* or in the TGF-beta pathway. MYH10 regulates actin organization, primary ciliary formation, and CN7 motor neuron migration.^25,39^ TGF-beta is expressed in motor neuron-adjacent mesenchyme^40^ and may act through a non cell-autonomous mechanism. We suggest additional studies of *MYH10* and TGF-beta genes in oCCDDs.

Our study has expanded the repertoire of variants in tubulin-encoding genes and identified multiple additional novel oCCDD candidate genes and variants, some of which had no prior human disease associations (e.g., *KLB, FGF21,* Supplementary Table 11). Future cross-phenotype examinations with other syndromic birth defect cohorts may solidify associations and identify additional shared etiologies, and ACMG/AMP/ClinGen and AlphaMissense classifications may prioritize the variants with highest potential.

Multiple factors likely contribute to the remainder of our cohort being unsolved. We used conservative classification criteria to mitigate overestimation of pathogenicity and only considered P/LP variants to fully or partially explain the phenotypes of the probands who harbored them. However, we identified many strong VUS candidates, some of which are particularly compelling and likely to become diagnostic over time (Table 1) or worthy of future novel candidate variant/gene research (Supplementary Table 11). Our cohort is phenotypically heterogeneous, suggesting that locus and allelic heterogeneity with frequent family-private variants could complicate identification of common genetic etiologies. This problem is not unique to oCCDDs, as exomes/genomes have exhausted large pedigrees and major genetic contributors to rare Mendelian conditions over time. (Fig. 3I). Additionally, since our study was restricted to coding variants, cases could be explained by other etiologies, including noncoding variants which remain challenging to interpret (https://doi.org/10.1101/2023.12.22.23300468). Finally, most probands had unilateral and sporadic oCCDDs, suggesting a lower likelihood of germline Mendelian genetic etiologies.

Limitations included bias toward genetically unsolved, syndromic, and sporadic cases; thus, our findings are not reflective of oCCDDs in the general population, many of which result from previously identified genetic etiologies. Moreover, because our cohort is predominantly of European descent, our study may miss other population-specific variants.

Our study expands the phenotypic spectrum of oCCDDs and elucidates missing genetic causes. This informs understanding of neurodevelopmental genetics and identifies novel genes and pathways to be prioritized in future studies.

## Supplementary Material

Supplement 1 Supplementary Table 1. Demographics and phenotypes of probands and affected relatives in the sequenced cohort

Supplement 2 Supplementary Table 5. Brain/orbital MRI findings

Supplement 3 Supplementary Table 6. SNVs/indels identified in pedigrees with at least 3 members sequenced

Supplement 4 Supplementary Table 7. Genetic findings in the cohort and genotype/phenotype correlations

Supplement 5 Supplementary Table 8. Candidate genes with putatively relevant animal models

Supplement 6 Supplementary Table 9. Gene ontology analysis of genes with de novo SNV/indels

Supplement 7 Supplementary Table 10. Structural variants identified in the cohort

Supplement 8

## Figures and Tables

**Table 1.** Prioritized genetic findings in the oCCDD cohort

Gene	Variant	ACMG/AMP/ ClinGen Classification	Category	Pedigrees	Diagnosis
**ACMG/AMP/ClinGen-Pathogenic and likely pathogenic variants**					
*KIF21A*	NP_001166935.1: p.(Arg954Trp)	P	[oCCDD+, Syndrome+/−]	198; ENG_AWA	Isolated sporadic CFEOM; isolated familial CFEOM
*ROBO3*	NP_071765.2: p.(Arg191ProfsTer61)	P	[oCCDD+, Syndrome+/−]	193	Syndromic sporadic HGP
*TUBB3*	NP_006077.2: p.(Asp417Asn)	P	[oCCDD+, Syndrome+/−]	ENG_ABW	Isolated sporadic CFEOM
*TUBB3*	NP_006077.2: p.(Gly71Arg)	P	[oCCDD+, Syndrome+/−]	81	Syndromic sporadic CFEOM
*ACTB*	NP_001092.1: p.(Ser348Leu)	P	[oCCDD(+), Syndrome+]	27	Syndromic sporadic congenital ptosis
*BBS1*	NP_078925.3: p.(Met390Arg)	P	[oCCDD(+), Syndrome+]	71	Syndromic sporadic CFEOM
*BBS1*	NP_078925.3: p.(Glu549Ter)	P	[oCCDD(+), Syndrome+]	71	Syndromic sporadic CFEOM
*DMD*	NP_003997.2: p.(Leu3485ArgfsTer11)	P	[oCCDD(+), Syndrome+]	227	Syndromic familial Brown syndrome
*EBF3*	NP_001362309.1: p.(Arg312Ter)	P	[oCCDD(+), Syndrome+]	42	Syndromic sporadic CN4-palsy
*EBF3*	NC_000014.9: g.53949639_56297420del	P	[oCCDD(+), Syndrome+]	233; 131	Syndromic sporadic DRS; syndromic sporadic DRS
*FGD1*	NP_004454.2: p.(Leu177ThrfsTer40)	P	[oCCDD(+), Syndrome+]	ENG_1894	Syndromic sporadic CFEOM
*GCH1*	NC_000014.9: g.53949639_56297420del	P	[oCCDD(+), Syndrome+]	ENG_BS	Syndromic familial congenital ptosis
*HDAC8*	NC_000023.11: g.72570670_72613916del	P	[oCCDD(+), Syndrome+]	13	Syndromic sporadic congenital ptosis
*KIAA0586*	NP_001316872.1: p.(Arg131LysfsTer4)	P	[oCCDD(+), Syndrome+]	ENG_AGZ	Syndromic sporadic CN6-palsy
*KIFBP*	NP_056449.1: p.(Ser200Ter)	P	[oCCDD(+), Syndrome+]	239	Syndromic familial CFEOM
*KMT2D*	NP_003473.3: p.(Arg5021Ter)	P	[oCCDD(+), Syndrome+]	128	Syndromic sporadic Brown syndrome
*MED13*	NP_005112.2:p.?	P	[oCCDD(+), Syndrome+]	61	Syndromic sporadic DRS
*PHOX2B*	NP_003915.2: p.(Ala256_Ala260dup)	P	[oCCDD(+), Syndrome+]	242	Syndromic sporadic MGJWS(+)ptosis
*PIEZO2*	NP_001365112.1: p.(Arg2799His)	P	[oCCDD(+), Syndrome+]	ENG_BAG	Syndromic sporadic CCDD-NOS
*PTPN11*	NP_002825.3: p.(Leu261Phe)	P	[oCCDD(+), Syndrome+]	ENG_CKA	Syndromic familial CCDD-NOS
*TRPV4*	NP_067638.3: p.(Arg269His)	P	[oCCDD(+), Syndrome+]	4	Syndromic sporadic CCDD-NOS
*ZC4H2*	NP_061154.1: p.(Lys81AsnfsTer6)	P	[oCCDD(+), Syndrome+]	144^16^	Syndromic sporadic DRS
*ARMC4*	NP_060546.2: p.(Ser892Ter)	P	[oCCDD-, Syndrome+]	269	Syndromic familial CFEOM
*CDK13*	NP_003709.3: p.(Gly717Arg)	P	[oCCDD-, Syndrome+]	ENG_2270	Syndromic sporadic DRS
*FOXG1*	NP_005240.3: p.(Gln86ArgfsTer106)	P	[oCCDD-, Syndrome+]	ENG_CHA	Syndromic sporadic CFEOM
*GJB2*	NP_003995.2: p.(Gly12ValfsTer2)	P	[oCCDD-, Syndrome+]	238	Syndromic familial DRS
*SCN1A*	NP_001159435.1: p.(Ile1545Val)	P	[oCCDD-, Syndrome+]	93	Syndromic sporadic DRS
*TGFB2*	NP_003229.1: p.(Arg299Trp)	P	[oCCDD-, Syndrome+]	ENG_FI	Isolated familial CCDD-NOS
*TUBA1A*	NP_006000.2: p.(His406Asp)	P	[oCCDD-, Syndrome+]	38^19^	Syndromic sporadic CFEOM
*FOXL2*	NP_075555.1: p.(Leu75Phe)	P	[Misdiagnoses]	ENG_JP	Isolated familial congenital ptosis
*ARMC9*	NP_001339683.2: p.(Thr293=)	LP	[oCCDD(+), Syndrome+]	269, 270	Syndromic familial CFEOM; syndromic sporadic CFEOM
*DYRK1A*	NP_001334650.1: p.(Leu66Ter)	LP	[oCCDD(+), Syndrome+]	26	Syndromic sporadic DRS
*FBN1*	NP_000129.3: p.(Cys1053Tyr)	LP	[oCCDD(+), Syndrome+]	ENG_ACR	Syndromic sporadic HGP
*KIAA0586*	NP_001316872.1: p.(Gln263Ter)	LP	[oCCDD(+), Syndrome+]	ENG_AGZ	Syndromic sporadic CN6-palsy
*KIFBP*	NP_056449.1: p.(Ala362SerfsTer8)	LP	[oCCDD(+), Syndrome+]	239	Syndromic familial CFEOM
*MED13*	NP_005112.2: p.(Leu1188IlefsTer9)	LP	[oCCDD(+), Syndrome+]	62	Syndromic sporadic DRS
*POGZ*	NP_055915.2: p.(Phe836LeufsTer18)	LP	[oCCDD(+), Syndrome+]	ENG_CMO	Syndromic sporadic congenital ptosis
*TGFBR2*	NP_003233.4: p.(Arg460Leu)	LP	[oCCDD(+), Syndrome+]	ENG_ADU	Syndromic sporadic HGP
*ZC4H2*	NP_061154.1: p.(Arg198Trp)	LP	[oCCDD(+), Syndrome+]	257	Syndromic sporadic DRS
*ZNF462*	NP_067047.4: p.(Arg255Ter)	LP	[oCCDD(+), Syndrome+]	48^17^	Syndromic familial congenital ptosis
*ZNF462*	NP_067047.4: p.(Tyr1704Ter)	LP	[oCCDD(+), Syndrome+]	ENG_AHO	Syndromic familial CFEOM
*CEP83*	NP_057206.2: p.(Glu530Ter)	LP	[oCCDD-, Syndrome+]	ENG_AZW	Syndromic sporadic CFEOM
*RYR1*	NP_000531.2: p.(Arg3772Trp)	LP	[Misdiagnoses]	ENG_AKG	Syndromic sporadic CFEOM
*TWIST1*	NP_000465.1: p.(Ala129_Ile135dup)	LP	[Misdiagnoses]	ENG_0640	Syndromic familial congenital ptosis
**Select ACMG/AMP/ClinGen-variants of uncertain significance with highest supportive evidence**					
*CHN1*	NP_001813.1: p.(Ala27Gly)	VUS	[oCCDD+, Syndrome+/−]	ENG_1580	Isolated sporadic DRS
*CHN1*	NP_001813.1: p.(Tyr21Cys)	VUS	[oCCDD+, Syndrome+/−]	ENG_BBG	Syndromic sporadic CN6-palsy
*MAFB*	NP_005452.2: p.(Glu223Lys)	VUS	[oCCDD+, Syndrome+/−]	232	Isolated familial DRS
*PHOX2A*	NP_005160.2: p.(Trp137Cys)	VUS	[oCCDD+, Syndrome+/−]	160	Syndromic sporadic CFEOM
*SALL4*	NC_000020.11: g.51783476_51785034del	VUS	[oCCDD+, Syndrome+/−]	DQ	Syndromic sporadic DRS
*ARMC9*	NP_001339683.2: p.(Arg343Ser)	VUS	[oCCDD(+), Syndrome+]	ENG_COX	Syndromic sporadic CFEOM
*COL25A1*	NP_942014.1: p.(Gly400Arg)	VUS	[oCCDD(+), Syndrome+]	56^20^	Syndromic sporadic CFEOM
*ECEL1*	NP_004817.2: p.(Cys772Arg)	VUS	[oCCDD(+), Syndrome+]	223	Syndromic familial DRS
*KIF21B*	NP_001239031.1: p.(Phe354Ser)	VUS	[oCCDD(+), Syndrome+]	ENG_FR	Syndromic sporadic CFEOM
*TUBB6*	NP_115914.1: p.(Glu410Lys)	VUS	[oCCDD(+), Syndrome+]	ENG_CML	Syndromic familial congenital ptosis
*MACF1*	NC_000001.11: g.39428731_39468326del	VUS	[oCCDD-, Syndrome+]	98^18^	Syndromic sporadic CN6-palsy
*TUBA1A*	NP_006000.2: p.(Ser379Asn)	VUS	[oCCDD-, Syndrome+]	170	Syndromic sporadic DRS
*TUBA4A*	NP_005991.1: p.(Arg390His)	VUS	[oCCDD-, Syndrome-]	ENG_IM	Isolated sporadic congenital ptosis
*CHRNE*	NP_000071.1: p.(Ile194Thr)	VUS	[Misdiagnoses]	ENG_2044	Syndromic sporadic congenital ptosis

## Data Availability

Exome/genome sequencing data are accessible under dbGaP accession numbers phs001247.v1.p1 and phs001272.v2.p1. SNVs, indels, and SVs that were newly ACMG/AMP/ClinGen-classified by our study were submitted to ClinVar (Accession IDs: SUB14307097, SUB14310205, SUB14279226). A subset of variants were previously interpreted by the ACMG/AMP criteria by our team and submitted to ClinVar under separate accession IDs (pedigree ENG_1580, SCV001445961.1; pedigree 42, SCV001445941.1; pedigree ENG_CMO, SCV003761257.1; pedigree 71, SCV002507051.1 and SCV000693896.1; pedigree 144, SCV001430799.1; pedigree 38, SCV001449530.1). A variant in pedigree 13 was previously interpreted by the Undiagnosed Diseases Network for the same individual as we report in our cohort (SCV000837689.1).

## Ocular CCDD Phenotyping Consortium

Hugo Abarca-Barriga, Christiane Al-Haddad, Jeffrey L. Berman, Erick D. Bothun, Jenina Capasso, Oscar Francisco Chacon-Camacho, Lan Chang, Stephen P. Christiansen, Maria Laura Ciccarelli, Monique Cordonnier, Gerald F. Cox, Cynthia J. Curry, Linda R. Dagi, Thomas Lee Dahm, Karen L. David, Bradley V. Davitt, Teresa De Berardinis, Joseph L. Demer, Julie Désir, Fabiana D’Esposito, Arlene V. Drack, Eric Eggenberger, James E. Elder, Alexandra T. Elliott, K. David Epley, Hagit Baris Feldman, Carlos R. Ferreira, Maree P. Flaherty, Anne B. Fulton, Christina Gerth-Kahlert, Irene Gottlob, Stephen Grill, Dorothy J. Halliday, Frank Hanisch, Eleanor Hay, Gena Heidary, Christopher Holder, Jonathan C. Horton, Alessandro Iannaccone, Sherwin J. Isenberg, Suzanne C. Johnston, Alon Kahana, James A. Katowitz, Melanie Kazlas, Natalie C. Kerr, Virginia Kimonis, Melissa W. Ko, Feray Koc, Dorte Ancher Larsen, Guillermo Lay-Son, Danielle M. Ledoux, Alex V. Levin, Richard L. Levy, Christopher J. Lyons, David A. Mackey, Adriano Magli, Iason S. Mantagos, Candice Marti, Isabelle Maystadt, Fiona McKenzie, Manoj P. Menezes, Claudia N. Mikail, David T. Miller, Kathryn Bisceglia Miller, Monte D. Mills, Kaori Miyana, H. U. Moller, Lisa Mullineaux, Julie K. Nishimura, A. Gwendolyn Noble, Pramod Kumar Pandey, Piero Pavone, Johann Penzien, Robert Petersen, James A. Phalen, Annapurna Poduri, Claudia R. Polo, Lev Prasov, Feliciano J. Ramos, Maria Ramos-Caceres, Richard M. Robb, Béatrice Rossillion, Mustafa Sahin, Harvey S. Singer, Lois E. H. Smith, Jeffrey A. Sorkin, Janet S. Soul, Sandra E. Staffieri, Heather J. Stalker, Steven F. Stasheff, Sonya Strassberg, Mitchell B. Strominger, Deepa Ajay Taranath, Ioan Talfryn Thomas, Elias I. Traboulsi, Maria Cristina Ugrin, Deborah K. VanderVeen, Andrea L. Vincent, Marlene C. Vogel G., Bettina Wabbels, Agnes M. F. Wong, C. Geoffrey Woods, Carolyn Wu, Edward Yang, Alison Yeung, Terri L. Young, Juan C. Zenteno, Alexandra A. Zubcov-Iwantscheff, Johan Zwaan
