# Supplementary material for: Expanding the genetics and phenotypes of ocular congenital cranial dysinnervation disorders
Source: medRxiv. 2024 Mar 26:2024.03.22.24304594. Preprint. [Version 1] doi: 10.1101/2024.03.22.24304594 (PMC10996726; doi:10.1101/2024.03.22.24304594)
Supplement: Supplement 8 [file NIHPP2024.03.22.24304594v1-supplement-8.pdf]

medRxiv preprint doi: https://doi.org/10.1101/2024.03.22.24304594; this version posted March 26, 2024. The copyright holder for this preprint (which was not certified by peer review) is the author/funder, who has granted medRxiv a license to display the preprint in perpetuity.
It is made available under a CC-BY 4.0 International license .

Index of Supplementary Materials, Methods, and Results

SUPPLEMENTARY METHODS **40**
Cohort enrollment, data collection, and phenotyping 40
Co-occurring defects analysis (CODA) of oCCDDs and syndromic phenotypes 44
DNA sample collection and sequencing 45
Genetic imputation of global ancestry 46
SNV/indel filtering and prioritization 47
Identification of recurrently mutated genes 47
Biological prioritization of SNVs/indels 48
DenovolyzeR analysis of de novo variants 48
Gene ontology analysis of DNVs 49
Coding structural variant (SV) analysis 50
Animal model analyses 51
Genotype/phenotype correlations 52
Interpretation of variant pathogenicity and submission to ClinVar 52
Variant categorization 53
Identification of recurrent missense variants 53
Evaluation of recurrent and prioritized missense variants with AlphaMissense 53
Figure generation 54
SUPPLEMENTARY RESULTS **55**
oCCDD Phenotype Summaries in Sequenced Probands 55
Syndromic/ Non-CCDD Phenotype Summaries 55
Brain MRI Findings in Sequenced Probands 56
Pre-existing genetic diagnoses 59
Analysis of genes from SNV/indel analyses and their animal model phenotypes 59
DenovolyzeR analysis of de novo SNVs/indels 62
Categorization of genes with ACMG/AMP/ClinGen-classifed SNVs/indels or SVs 63
SUPPLEMENTARY REFERENCES **66**
SUPPLEMENTARY WEB RESOURCES **69**
SUPPLEMENTARY FIGURES **70**
Supplementary Figure 1. Human cranial nerve schematics 70
Supplementary Figure 2. Sequencing type among 467 probands 72
Supplementary Figure 3. Sequencing structure among 467 probands 72
Supplementary Figure 4. Sporadic versus familial oCCDDs among 467 probands 73
Supplementary Figure 5. Syndromic versus isolated oCCDD breakdown among 467 probands 73
Supplementary Figure 6. Combined sporadic/familial and isolated/syndromic oCCDD breakdown among 467 probands 74
Supplementary Figure 7. Laterality of oCCDDs among 467 probands 74
Supplementary Figure 8. Documented synkinesis among 467 probands 75

medRxiv preprint doi: https://doi.org/10.1101/2024.03.22.24304594; this version posted March 26, 2024. The copyright holder for this preprint (which was not certified by peer review) is the author/funder, who has granted medRxiv a license to display the preprint in perpetuity.
It is made available under a CC-BY 4.0 International license .

Supplementary Figure 9. Protein mapping of MYH10 variants identified in our cohort and reported in the literature 75
Supplementary Figure 10. Characteristics among pedigrees with ACMG/AMP/ClinGen-P/LP variants 76
SUPPLEMENTARY TABLES 77
Supplementary Table 2. Sequencing, demographics, and oCCDD information among 467 probands 77
Supplementary Table 3. Syndromic features among the 197 probands with syndromic oCCDDs 78
Supplementary Table 4. CODA analysis of co-occurring syndromic phenotypes 79
Supplementary Table 11. Additional novel oCCDD candidate genes/variants of uncertain significance that may merit additional study 83

NOTE-The following additional Supplementary Tables were generated but are too large to include in the present file. These are uploaded as separate supplementary spreadsheets:
Supplementary Table 1. Demographics and phenotypes of probands and affected relatives in the sequenced cohort
Supplementary Table 5. Brain/orbital MRI findings
Supplementary Table 6. SNVs/indels identified in pedigrees with at least 3 members sequenced
Supplementary Table 7. Genetic findings in the cohort and genotype/phenotype correlations
Supplementary Table 8. Candidate genes with putatively relevant animal models
Supplementary Table 9. Gene ontology analysis of genes with de novo SNV/indels
Supplementary Table 10. Structural variants identified in the cohort

medRxiv preprint doi: https://doi.org/10.1101/2024.03.22.24304594; this version posted March 26, 2024. The copyright holder for this preprint (which was not certified by peer review) is the author/funder, who has granted medRxiv a license to display the preprint in perpetuity.
It is made available under a CC-BY 4.0 International license .

SUPPLEMENTARY METHODS

Cohort enrollment, data collection, and phenotyping

This study of 467 pedigrees was derived from a large cohort of individuals with oCCDDs and their relatives enrolled in our research study at Boston Children's Hospital from August 1992 through June 2019. Individuals with the following oCCDD diagnoses were included: CFEOM, ptosis, MGJWS(+)ptosis, MGJWS(-)ptosis, INV-MGJWS(-)ptosis, CN4-palsy, Brown syndrome, DRS, CN6-palsy, HGP, or CCDD-NOS. Individuals with Moebius syndrome were not included in the present study. This study was conducted in accordance with the Declaration of Helsinki and approved by the Institutional Review Board of Boston Children's Hospital (BCH), Boston, MA (protocol 05-03-036R). Data were collected in accordance with the ethical guidelines of BCH. Written informed consent was obtained by qualified individuals for study participation. Participants' genomic and clinical data remained linked but were deidentified.

Many but not all study participants (403/467 probands; 86.3%) were pre-screened for pathogenic variants in reported oCCDD disease genes (*KIF21A, PHOX2A, TUBB3, TUBB2B, CHN1, MAFB, SALL4, HOXA1*, *ROBO3*, and/or *ACKR3*) if their oCCDD phenotype described on initial enrollment was consistent with the phenotype attributed to the oCCDD gene. Screening approaches varied based on available technologies at the time but included exome sequencing and Sanger sequencing validation, Sanger sequencing, ddPCR, Fluidigm, Haloplex, long-range PCR, qPCR, DHPLC, Surveyor, and SSCP. Pedigrees qualified for exome sequencing or genome sequencing (ES/GS) if they screened negative for common genetic etiologies, had sufficient DNA quality and quantity, and consented to broad genomic data sharing. Among these, pedigrees were prioritized if the oCCDD was familial, if the phenotype included syndromic features, and if the proband had been screened for variants in the common oCCDD genes.

medRxiv preprint doi: https://doi.org/10.1101/2024.03.22.24304594; this version posted March 26, 2024. The copyright holder for this preprint (which was not certified by peer review) is the author/funder, who has granted medRxiv a license to display the preprint in perpetuity.
It is made available under a CC-BY 4.0 International license .

Demographics including race, ethnicity, and biological sex assigned at birth were collected via survey and self-reported by study participants or their parents/legal guardians. These data were collected as required by the US National Institutes of Health and serve to contextualize the generalizability and limitations of our findings. Biological sex assigned at birth is reported at the individual and cohort levels, and race and ethnicity are reported at the cohort level only. Self-reported categories of race included American Indian or Alaska Native, Asian, Black / African American, Native Hawaiian / Other Pacific Islander, More Than One Race, or White per the NIH Policy on Reporting Race and Ethnicity Data. Self-reported ethnicities included Hispanic or Latino, and Non-Hispanic or Latino.

Phenotypic data were obtained through retrospective review of clinical records, questionnaires, and clinical updates from research participants and their clinicians. Some probands self-acquired and shared ocular motility photographs using StrabisPIX, a HIPAA-compliant ophthalmologic application. When available, ocular motility data, photographs, and videos were reviewed retrospectively by a team of ophthalmologists, orthoptists, and neurologists (authors DGH, MCW, SM, ECE).

Affected individuals were assessed for presence or absence of phenotypes in 20 broad categories: restricted vertical eye movement, eyelid ptosis, restricted horizontal eye movement, synkinetic eye/facial movements, facial paralysis, hearing impairment, lower cranial nerve abnormalities (CN IX-XII), central nervous system (CNS) structural/functional malformation, malformations or dysfunction of additional peripheral nervous system (PNS)/muscle/connective tissue, craniofacial dysmorphisms, non-craniofacial dysmorphisms, skeletal abnormalities (non-scoliosis), skeletal abnormalities (scoliosis), pulmonary/lung/respiratory abnormalities, cardiovascular abnormalities, gastrointestinal (GI) abnormalities, renal/urinary/genital abnormalities, endocrine abnormalities, skin/hair/teeth/nail abnormalities, and other

medRxiv preprint doi: https://doi.org/10.1101/2024.03.22.24304594; this version posted March 26, 2024. The copyright holder for this preprint (which was not certified by peer review) is the author/funder, who has granted medRxiv a license to display the preprint in perpetuity. It is made available under a CC-BY 4.0 International license .

dysmorphisms/involvement. Data were not available for all categories for all participants.

When available, brain magnetic resonance images (MRIs) were obtained clinically and reviewed retrospectively by a pediatric neuroradiologist (author CR) for cranial nerve/ extraocular muscle findings and imaging quality, structural brain abnormalities, and abnormalities in other features including the orbits, globes, muscles of mastication, facial muscles, temporal bones, inner ear, internal auditory canals, skull, craniofacial shape, and spine. MRIs obtained by 3T imaging with thin slices (~1 mm) were considered optimized for cranial nerve / extraocular muscle detection. When available, other imaging such as spinal MRIs or magnetic resonance angiography were reviewed for additional anomalies of the spine or vasculature, respectively. CN 1-12 were each read as normal, thin, absent/not visualized, or abnormal trajectory. Visible extraocular muscles were assessed for presence/ absence, size, and shape. Brain structures were assessed including cortex, corpus callosum, anterior commissure, basal ganglia, hippocampi, olfactory system, cerebral ventricles, white matter, myelination, cerebellar hemispheres, cerebellar vermis, midbrain, pons, and medulla.

In most pedigrees, the proband was designated as the individual in the pedigree who was first referred to our research team. In 6 pedigrees (ENG_CMV, ENG_CKA, ENG_VC, ENG_0686, ENG_1211, and ENG_2228), DNA from the true proband was not sequenced, and thus a secondary affected individual whose DNA was sequenced was designated as the proband. Probands and pedigrees were assigned oCCDD diagnoses based on the oCCDD present in the proband. Mixed intrafamilial oCCDD presentations and oCCDD laterality for each proband and affected individual are noted (Supplementary Table 1).

medRxiv preprint doi: https://doi.org/10.1101/2024.03.22.24304594; this version posted March 26, 2024. The copyright holder for this preprint (which was not certified by peer review) is the author/funder, who has granted medRxiv a license to display the preprint in perpetuity.
It is made available under a CC-BY 4.0 International license .

Individuals were categorized as isolated if they had an oCCDD but no additional non-ocular congenital findings. Individuals were categorized as syndromic if they had convincing documentation of at least one major or two minor congenital anomalies in addition to their oCCDD and/or if clinical genetic evaluation included chromosomal/genetic analysis for syndromic features. Major and minor congenital anomalies were defined using the CDC Birth Defects Surveillance Toolkit.[1] Scoliosis was counted as a major anomaly if it required medical intervention. Hearing impairment was counted as a major anomaly if attributable to non-conductive etiologies, including sensorineural deficits or congenital ear malformations. Syndromic findings that were explained by an alternative genetic diagnosis are denoted.

Individuals who lacked a family history of oCCDDs or related findings were categorized as sporadic. Individuals with a family history of oCCDDs were designated as familial. Pedigree structures were assessed to determine the most likely mode(s) of inheritance.

If both the proband and one or more relatives had a syndromic oCCDD, the pedigree was designated as “syndromic familial” and both the oCCDD and syndromic features were documented as the heritable components in the pedigrees. If, however, the proband had a syndromic oCCDD and had relatives with the syndromic features in the absence of the oCCDD, the pedigree was designated as “syndromic familial” and the non-CCDD phenotype was documented as the heritable component in the pedigree (Supplementary Table 1). In such pedigrees, only the proband was designated as “affected” for counting purposes, since only the proband had an oCCDD. Pedigrees were designated as “isolated” if syndromic feature(s) were absent in the proband and present in family members who did not have oCCDDs.

medRxiv preprint doi: https://doi.org/10.1101/2024.03.22.24304594; this version posted March 26, 2024. The copyright holder for this preprint (which was not certified by peer review) is the author/funder, who has granted medRxiv a license to display the preprint in perpetuity.
It is made available under a CC-BY 4.0 International license .

For pedigrees with highlighted genetic findings, individual clinical features were reported using Human Phenotype Ontology (HPO) terms.[2] Certain findings were noted clinically but not included as separate HPO terms if these findings are frequently encompassed by the overarching oCCDD diagnosis (e.g., esotropia, exotropia, amblyopia, and ptosis frequently accompany CFEOM). Highlighted cases were assessed for correlative phenotypic data to obtain supportive evidence for or against candidate gene/variant causality.

Co-occurring defects analysis (CODA) of oCCDDs and syndromic phenotypes

Co-occurring defects analysis (CODA) was used to determine whether oCCDDs and syndromic phenotypes co-occurred more frequently than by chance.[3] Separate analyses of syndromic phenotypes were performed for each of the most common oCCDD subdiagnoses (DRS, CFEOM, and congenital ptosis). CODA examines the observed number of cases for all combinations of two through five co-occurring phenotypes and determines whether each combination occurs more frequently than if the phenotypes were independent. The method adjusts for the tendency of birth defects to co-occur with other birth defects and produces an adjusted observed/expected ratio (OEadj). OEadj values >1, which indicate enrichment of the co-occurrence of the given phenotypes, were retained if present in at least 3 probands for DRS (n=79 total syndromic probands), at least 3 probands for CFEOM (n=50 total syndromic probands), or at least 2 probands for congenital ptosis (n=26 total syndromic probands). Partially redundant phenotype combinations were removed. Syndromic phenotypes used for CODA included 15 of the 20 syndromic categories defined in the “Cohort enrollment, data collection, and phenotyping” section above. The miscellaneous “other findings" category of phenotypes was excluded due to large amounts of missing data. Four of the 20 syndromic categories were also excluded since they define specific oCCDD subcategories and thus provide less informative associations: restricted vertical motility, restricted horizontal motility, synkinesis, and ptosis.

medRxiv preprint doi: https://doi.org/10.1101/2024.03.22.24304594; this version posted March 26, 2024. The copyright holder for this preprint (which was not certified by peer review) is the author/funder, who has granted medRxiv a license to display the preprint in perpetuity.
It is made available under a CC-BY 4.0 International license .

DNA sample collection and sequencing

Of 1310 genetically unsolved pedigrees in our cohort, 467 underwent exome/genome sequencing and phenotypic analyses for this study (550 affected and 1108 total individuals). DNA was extracted from blood or saliva of oCCDD probands and their relatives using Puregene manual blood extraction or prepIT.L2P saliva DNA extraction solution (DNA Genotek, Ottawa, ON, Canada). Each pedigree underwent exome or genome sequencing. Exome sequencing was conducted through the Center for Mendelian Genomics (CMG) at the Eli and Edythe L. Broad Institute of MIT and Harvard (Broad CMG; Cambridge, MA, USA). Genome sequencing was conducted through the Broad CMG or through the NIH Gabriella Miller Kids First (GMKF) Pediatric Research Program at the Baylor College of Medicine Human Genome Sequencing Center (Houston, TX) and then reprocessed by the Broad CMG. We refer to these sequencing datasets as CMG_ES, CMG_GS, and GMKF_GS, respectively.

The three datasets were generated at different times through separate collaborative initiatives. As such, each had unique selection criteria that determined whether samples underwent exome or genome sequencing. The first dataset sequenced was GMKF_GS, which prioritized pedigrees for genome sequencing for which adequate DNA and data sharing consents were available for at least 3 pedigree members. In addition, pedigrees with syndromic oCCDDs were prioritized to search for overlap with other developmental phenotypes. The second genomed dataset was CMG_GS, which permitted genome sequencing of a smaller number of samples and was aimed at identifying additional alleles in candidate genes identified in familial pedigrees sequenced through GMKF_GS. Accordingly, this dataset was predominantly composed of singletons with oCCDDs and a few additional larger pedigrees with adequate DNA and consents. The final dataset generated was CMG_ES, which permitted exome sequencing of a larger number of samples with variable phenotypes and pedigree structures that had adequate DNA and consents. Consequently, this cohort included heterogeneous samples that had not

medRxiv preprint doi: https://doi.org/10.1101/2024.03.22.24304594; this version posted March 26, 2024. The copyright holder for this preprint (which was not certified by peer review) is the author/funder, who has granted medRxiv a license to display the preprint in perpetuity.
It is made available under a CC-BY 4.0 International license .

been sequenced in either of the aforementioned genome sequencing datasets.

DNA libraries were generated for CMG_ES with a 38-Mb target Illumina exome capture and sequenced with 150 bp paired-end reads to cover >80% of targets at 20x, with an average target coverage >55x. DNA libraries were prepared for GMKF_GS with the KAPA Hyper PCR-free library prep kit (KAPA Biosystems Inc., Wilmington, MA) and sequenced on the Illumina HiSeq X to 30X average coverage using 150 bp paired-end reads. PCR-free DNA libraries were made for CMG_GS using Illumina HiSeq X Ten v2 and sequenced with an average target coverage >30x. Quality assurance checks were performed to confirm sample identity and parentage when appropriate. All datasets were then processed at the Broad using a Picard-based pipeline (http://broadinstitute.github.io/picard/) integrating base quality score recalibration and local realignment around indels. Reads were mapped to the human reference genome (GRCh38)[4] using BWA.[5] Single nucleotide variants (SNVs) and small insertions and deletions <50bp (indels) were called jointly among >10,000 exomes or genomes assembled by the Broad using Genome Analysis Toolkit (GATK) HaplotypeCaller v4.0 (GMKF_GS and CMG_GS) or v3.4 (CMG_ES).[6,7] SNVs and indels were filtered using default GATK Variant Quality Score Recalibration parameters. Variants were annotated with Ensembl Variant Effect Predictor[8] and analyzed with seqr (https://seqr.broadinstitute.org/).[9]

Genetic imputation of global ancestry

Global genetic ancestry was inferred from exome/genome sequences using principal component analysis as previously described.[10] Individuals were classified into the following genetically imputed ancestral groups: African/ African American, Amish, Ashkenazi Jewish, East Asian, European (Finnish), European (non-Finnish), Latino/Admixed American, Remaining Individuals, or South Asian. These ancestral genetic groups were reported at an aggregate level for the cohort.

medRxiv preprint doi: https://doi.org/10.1101/2024.03.22.24304594; this version posted March 26, 2024. The copyright holder for this preprint (which was not certified by peer review) is the author/funder, who has granted medRxiv a license to display the preprint in perpetuity.
It is made available under a CC-BY 4.0 International license .

SNV/indel filtering and prioritization

For each pedigree, SNVs and indels were analyzed according to the most probable mode(s) of inheritance based on pedigree structure using seqr (https://seqr.broadinstitute.org/).[9] This included custom analyses to account for observed incomplete penetrance, when present. Using 1000 Genomes Project Phase 3,[11] ExAC v0.3,[12] gnomAD v2.0.2,[10] and TOPMed Freeze 5[13] reference datasets, we identified homozygous or compound heterozygous variants with allele frequencies (AF) <0.01 under an autosomal recessive (AR) model or heterozygous variants with AF<0.001 under autosomal dominant (AD), *de novo*, or X-linked recessive (XLR) models. Variants that were homozygous in reference population(s) were excluded. Heterozygous variants present in >5 individuals and all variants with AF>0.01 in an internal database of rare disease samples from the Broad CMG were excluded. Variants that passed quality control filters and had allele balances >25 and genotype qualities >20 were selected. Indels and missense, nonsense, or essential (+/-2 bp) splice site-altering variants were prioritized in all annotated protein-coding genes. Broader noncoding variant analyses were conducted and reported separately (https://doi.org/10.1101/2023.12.22.23300468). Variant annotation was confirmed in June of 2023 with VariantValidator.[14] MANE Select transcripts were prioritized. Lists of all variants meeting these parameters in singletons were generated, but singleton sequences were assessed only for variants in known oCCDD genes or strong candidate genes and for variants annotated as pathogenic/ likely pathogenic in ClinVar in additional genes.

Identification of recurrently mutated genes

Genes that had SNVs/indels meeting the parameters defined in “SNV/indel filtering and prioritization” that were mutated in more than one pedigree were defined as “recurrently mutated genes.” Recurrently mutated genes were queried in all 467 pedigrees regardless of specific oCCDD diagnosis.

medRxiv preprint doi: https://doi.org/10.1101/2024.03.22.24304594; this version posted March 26, 2024. The copyright holder for this preprint (which was not certified by peer review) is the author/funder, who has granted medRxiv a license to display the preprint in perpetuity.
It is made available under a CC-BY 4.0 International license .

Biological prioritization of SNVs/indels

Further qualitative prioritization was performed of candidate variants based on their CADD v1.6 PHRED scores.[15] Candidate genes were further prioritized based on their LOEUF[10] and missense z-scores;[16] functions annotated in the primary literature, and prior reported associations with human phenotypes in Online Mendelian Inheritance in Man (OMIM, www.omim.org),[17] the Human Gene Mutation Database (HGMD, www.hgmd.cf.ac.uk; license #7597755),[18] and ClinVar[19] databases and in the primary literature. Select variants were prioritized based on: prior reporting in unrelated individuals in the literature or in ClinVar; consistency of the phenotypes in our proband(s) relative to previously reported probands with variants in the same gene; or of identification of multiple rare, putatively damaging alleles in unrelated individuals with similar phenotypes in our cohort.

DenovolyzeR analysis of de novo variants

Statistical analysis of SNV/indel de novo variants (DNVs) was performed with DenovolyzeR.[20] 203 pedigrees were amenable to DNV analysis and comprised a minimum structure of 2 unaffected parents and 1 affected child (129 from GMKF_GS; 6 from CMG_GS; 68 from CMG_ES). If additional relatives were also sequenced outside of the nuclear trio, these members' sequences were omitted. Pedigrees with 2 affected siblings of two unaffected parents were excluded from the analysis, thus omitting pedigrees with putative germline mosaicism. In total, 197 trios, 4 quads, and 2 pedigrees with other pedigree structures (>4 sequenced individuals) were used. The individuals whose sequences were used for these analyses are specified (Supplementary Table 1, Column I). oCCDDs in these pedigrees were variable (51 isolated DRS, 36 syndromic DRS, 12 isolated CFEOM, 28 syndromic CFEOM, 9 isolated congenital ptosis, 11 syndromic congenital ptosis, 13 isolated MGJWS(+)ptosis, 5 syndromic MGJWS(+)ptosis, 3 isolated MGJWS(-)ptosis, 2 syndromic MGJWS(-)ptosis, 1 isolated INV-MGJWS(-)ptosis, 11 isolated CN4-palsy, 10 syndromic CN4-palsy, 2 isolated Brown

medRxiv preprint doi: https://doi.org/10.1101/2024.03.22.24304594; this version posted March 26, 2024. The copyright holder for this preprint (which was not certified by peer review) is the author/funder, who has granted medRxiv a license to display the preprint in perpetuity.
It is made available under a CC-BY 4.0 International license .

syndrome, 2 syndromic Brown syndrome, 3 syndromic horizontal gaze palsy, 2 syndromic CN6-palsy, 2 syndromic CCDD-NOS).

Heterozygous autosomal missense, nonsense, canonical splice site, synonymous, and frameshifting SNVs/indels were selected that occurred de novo in the proband and had AF<0.001 in the 1000 Genomes Project Phase 3,[11] ExAC v0.3,[12] gnomAD v2.0.2,[10] and TOPMed Freeze 5[13] databases and <0.01 in an internal database of rare disease samples. Variants were retained that passed filters and had genotype quality >30, allele balance >25, read depth >10, and alternate allele count ≥5. Three pedigrees (67, 252, and 14) had DNV counts > 3 SD from the mean and were considered outliers and excluded from further analysis.

Variants were aggregated into classes based on predicted loss-of-function (pLOF; nonsense, splicing, frameshift) or missense consequences. If multiple DNVs of any class were represented more than once in a single proband (e.g., 2 missense variants in the same gene in one person), only one variant was counted for downstream analyses. Through denovolyzeR, we used the functions denovolyzeByClass to assess overall enrichment of various classes of DNVs, denovolyzeMultiHits to assess enrichment of recurrently mutated genes with DNVs in any class, and denovolyzeByGene to assess whether enrichment within any class was attributable to recurrent DNVs in any individual genes as opposed to collective DNVs across multiple genes.

Gene ontology analysis of DNVs

DNVs were obtained for gene ontology (GO) analysis as described in the denovolyzeR methods above, and synonymous variants were removed. Functional enrichment analysis was performed using g:Profiler g:GOSt (version e110_eg57_p18_4b54a898) with g:SCS multiple testing correction and a significance threshold of 0.05.[21] P-values are provided as adjusted p-values (p_{adj}) obtained after multiple test correction.

medRxiv preprint doi: https://doi.org/10.1101/2024.03.22.24304594; this version posted March 26, 2024. The copyright holder for this preprint (which was not certified by peer review) is the author/funder, who has granted medRxiv a license to display the preprint in perpetuity.
It is made available under a CC-BY 4.0 International license .

Coding structural variant (SV) analysis

In samples with genome sequencing, structural variants (SVs) that perturb the coding sequence were identified, jointly genotyped, and annotated using the ensemble SV discovery tool GATK-SV (https://github.com/broadinstitute/gatk-sv).[22] Each cohort was processed with samples from pedigrees with unrelated rare disorders to serve as controls for improved filtering. SVs in each oCCDD pedigree were analyzed according to the most probable mode(s) of inheritance based on pedigree structure. *De novo* SVs were detected using a published pipeline that applies a series of post-hoc filters to a GATK-SV VCF.[23] Rare inherited SVs were identified by filtering on an AF<0.005 based on unaffected individuals in our internal cohort. In addition, the gnomAD SVs v2.1 database was used for AF filtering using the same cutoffs defined for SNVs/indels. Partial or full deletions of ≥1 exon and duplication or inversion events with exonic breakpoint(s) were prioritized for pathogenicity review. SVs were visually inspected with IGV[24] and the RdTest module in GATK-SV for validation, and passing variants were prioritized. Genes within each SV interval were evaluated for predicted haploinsufficiency or triplosensitivity[25] and biological relevance, recurrent disruption of the same gene(s) among multiple pedigrees, and putatively relevant animal models, as defined in “animal model analyses” described below.

In samples with exome sequencing, SVs consisting of rare coding deletions and duplications were delineated with the recently published GATK-gCNV algorithm.[26] GATK-gCNV is a read-depth based method, specifically tailored to detect rare coding copy number variants (CNVs) from ES data with excellent sensitivity and false positive identification characteristics. In brief, we batched samples to be processed across different technical cohorts based on technical sequencing fluctuations such as those arising from a difference in sequencing center or in exome enrichment kits. Each of these technical cohorts was then relayed into the GATK-gCNV pipeline, which automatically normalized the read-depth signal onto the copy number scale and

medRxiv preprint doi: https://doi.org/10.1101/2024.03.22.24304594; this version posted March 26, 2024. The copyright holder for this preprint (which was not certified by peer review) is the author/funder, who has granted medRxiv a license to display the preprint in perpetuity.
It is made available under a CC-BY 4.0 International license .

output highly sensitive candidate CNVs. A series of deeply benchmarked quality control filtering metrics, as outlined in our recent publication,[27] was applied to prioritize CNVs. *De novo* status of these identified CNVs was determined in settings where we had complete trios by examining whether either of the parents harbored evidence of a matching CNV as the proband. Likewise, as with the samples with genome sequencing, genes within the identified CNVs were evaluated for predicted biological relevance, recurrent disruption of the same gene(s) among multiple pedigrees, and putatively relevant animal models, as defined in “animal model analyses” below.

Animal model analyses

Using the thresholds for allele frequency, variant annotations, and quality control defined above in the “SNV/indel filtering and prioritization” and “Coding structural variant (SV) analysis” sections, recurrently mutated genes as well as strong candidate genes meeting criteria defined in “Biological prioritization of SNVs/indels” were annotated for putatively relevant animal model phenotypes in the Monarch database (http://monarchinitiative.org).[28] Putatively relevant phenotypic terms were extremely diverse; examples included phenotypes in cranial nerves, motor neurons, other neurodevelopmental phenotypes, abnormalities of the orbital region, putatively relevant biological processes (e.g. involvement in axon growth/ guidance), and other syndromic features that are seen in some oCCDD cases (e.g. limb anomalies). We performed this analysis while bearing in mind the caveat that most animal models harbor loss-of-function alleles, while others have specific resulting in gain-of-function or altered function alleles, and these allelic consequences may or may not be consistent with the consequences of the variant(s) in our sequenced human cohort.

Following the identification of all genes harboring candidate variants that had compelling animal model data, we next evaluated each for full-gene and local missense constraint, predicted variant pathogenicity, population frequency, protein domain localization, relative location of

medRxiv preprint doi: https://doi.org/10.1101/2024.03.22.24304594; this version posted March 26, 2024. The copyright holder for this preprint (which was not certified by peer review) is the author/funder, who has granted medRxiv a license to display the preprint in perpetuity.
It is made available under a CC-BY 4.0 International license .

pedigrees' variants, and biological function. Human probands with variants in the same gene were evaluated for phenotypic consistency with one another and with their animal model orthologs. Genes/variants that were most compelling based on these collective criteria were highlighted.

Genotype/phenotype correlations

For pedigrees with highlighted genetic findings obtained through analyses of genotype/phenotypes, animal models, and/or DNVs, clinical features were reported using Human Phenotype Ontology (HPO).[2] Highlighted cases were assessed for correlative phenotypic data to obtain supportive evidence for or against candidate gene/variant causality.

Interpretation of variant pathogenicity and submission to ClinVar

Through qualitative manual review of genes obtained from analysis of genotype/phenotypes, DNVs, and animal models, select variants were chosen for formal interpretation. SNVs and indels were interpreted in May of 2023 using recommendations from the American College of Genetics and Genomics and Association for Molecular Pathology (ACMG/AMP)[29] in accordance with the standard operating procedure defined by the Clinical Genome Resource (ClinGen) Variant Curation Committee (ClinGen General Sequence Variant Curation Process Version 1.0; https://www.clinicalgenome.org/site/assets/files/3677/clingen_variant-curation_sopv1.pdf), using REVEL as the computational predictor for missense variation.[30] SVs were interpreted in May of 2023 using joint recommendations from ACMG and the Clinical Genome Resource (ClinGen).[31] The number of genes within each SV interval was annotated using OMIM. Variants were classified as pathogenic (P), likely pathogenic (LP), or variants of uncertain significance (VUS). A subset of variants were previously interpreted by the ACMG/AMP criteria by our team and submitted to ClinVar under separate accession IDs (Data Availability). Variant annotation was standardized using VariantValidator.[14]

medRxiv preprint doi: https://doi.org/10.1101/2024.03.22.24304594; this version posted March 26, 2024. The copyright holder for this preprint (which was not certified by peer review) is the author/funder, who has granted medRxiv a license to display the preprint in perpetuity.
It is made available under a CC-BY 4.0 International license .

Variant categorization

The genes that harbored ACMG/AMP/ClinGen-classified SNVs/indels or SVs were grouped into five categories as detailed in main text and Figure 3:

1) [oCCDD+,Syndrome+/-]: genes that were definitively associated with oCCDDs before this study and were genetically pre-screened in most probands.
2) [oCCDD(+),Syndrome+]: genes that had at least occasional prior oCCDD association but were typically part of specific monogenic syndromes and thus not pre-screened.
3) [oCCDD-,Syndrome+]: genes that fit the syndromic component of each proband's phenotype but that, to our knowledge, have no prior oCCDD association.
4) [oCCDD-,Syndrome-]: genes that, to our knowledge, had no reported association with either the oCCDD or non-CCDD phenotype of the probands who harbor them.
5) [Misdiagnoses]: genes associated with alternative non-neurogenic/ non-CCDD etiologies and represent misdiagnoses or oCCDD phenocopies.

Identification of recurrent missense variants

Using the thresholds for allele frequency, variant annotations, and quality control defined above in the "SNV/indel filtering and prioritization" section, recurrent heterozygous variants were identified among DNVs in sporadic cases, autosomal dominant variants in familial cases, and heterozygous variants in duos/singletons. Variants were considered recurrent if mutated among >1 pedigree with any oCCDD subdiagnosis, not just among pedigrees with the same oCCDD. Recurrent missense variants were not prioritized for ACMG/AMP classification, but were scored by AlphaMissense, as defined in the following section.

Evaluation of recurrent and prioritized missense variants with AlphaMissense

We scored both recurrent missense variants and ACMG/AMP-classified missense variants using AlphaMissense.[32] The latter included all heterozygous, homozygous, or compound

medRxiv preprint doi: https://doi.org/10.1101/2024.03.22.24304594; this version posted March 26, 2024. The copyright holder for this preprint (which was not certified by peer review) is the author/funder, who has granted medRxiv a license to display the preprint in perpetuity.
It is made available under a CC-BY 4.0 International license .

heterozygous missense variants that were represented in AlphaMissense and classified as pathogenic, likely pathogenic, or VUS by ACMG/AMP criteria (Supplementary Table 7). ACMG/AMP/ClinGen-VUS that we felt were most compelling and featured in Table 1 were distinguished from additional VUS that currently have lower levels of evidence.

AlphaMissense predictions were based on default score cutoffs designed to reach 90% precision on ClinVar variants. Variants evaluated by AlphaMissense were concurrently stratified into the five categories described above to assess breakdown of AlphaMissense scores within each category. Additionally, AlphaMissense scores were compared to ACMG/AMP classifications.

Figure generation

Components of figures were generated in R, and main and supplementary figures were assembled with BioRender.com using an academic license provided to Boston Children's Hospital (figure license numbers: KM26LVQ8FB, QW26LVQ72D, FK26LVQ58S, YX26JQRKIF, CM26JQRIAP, AI26JQUI4A, JC26JQWDZS, VH26JQW1Z0, UZ26JQWNGK, OQ26KR14PB, BU26JQX3RC).

medRxiv preprint doi: https://doi.org/10.1101/2024.03.22.24304594; this version posted March 26, 2024. The copyright holder for this preprint (which was not certified by peer review) is the author/funder, who has granted medRxiv a license to display the preprint in perpetuity.
It is made available under a CC-BY 4.0 International license .

SUPPLEMENTARY RESULTS

oCCDD Phenotype Summaries in Sequenced Probands

Multiple oCCDDs were occasionally seen within a single pedigree (n=7, 1.5%). In 4 syndromic DRS pedigrees (0.9%), the syndromic non-CCDD component of the phenotype was inherited, but the oCCDD was present only in 1 affected individual in the pedigree (Supplementary Table 1). By subdiagnoses, oCCDDs were unilateral in 62.6% of DRS, 38.8% of CFEOM, 73.3% of congenital ptosis, 73.6% of MGJWS, 67.9% of CN4-palsy, 69.2% of Brown syndrome, 0% of horizontal gaze palsy, 40% of CN6-palsy, and 44.4% of CCDD-NOS probands (Fig. 2B, Supplementary Fig. 7, Supplementary Tables 1-2). In total, 284 of 467 probands (60.8%) had some form of synkinesis (Fig. 2C). By definition, this includes all probands with DRS and MGJWS. Synkinesis was also common in CCDD-NOS (88.9%) and CFEOM (28.8%), and less frequent in Brown syndrome (7.7%) and congenital ptosis without MGJWS (1.3%). Synkinesis was not documented in probands with CN4-palsy, horizontal gaze palsy, or CN6-palsy (Supplementary Fig. 8, Supplementary Tables 1-2). Synkinetic patterns varied with diagnosis and among probands (Supplementary Table 1).

Syndromic/ Non-CCDD Phenotype Summaries

Among the 197 probands with a syndromic oCCDD, their syndromic findings were categorized as: CNS (55%), PNS/muscle/connective tissue (37%), craniofacial (37%), skeletal (24%), gastrointestinal (23%), cardiovascular (22%), skin/hair/teeth/nails (22%), other dysmorphisms (19%), renal/urinary/genital (17%), hearing (13%), endocrine (13%), scoliosis (12%), pulmonary/lung/respiratory (12%), facial paralysis (8%), and lower CN IX-XII (5%); the distribution of these syndromic findings were variable among oCCDD subdiagnoses (Fig. 2D, Supplementary Tables 1 and 3).

CODA analysis of co-occurring phenotypes among syndromic probands with DRS, CFEOM, or

medRxiv preprint doi: https://doi.org/10.1101/2024.03.22.24304594; this version posted March 26, 2024. The copyright holder for this preprint (which was not certified by peer review) is the author/funder, who has granted medRxiv a license to display the preprint in perpetuity.
It is made available under a CC-BY 4.0 International license .

congenital ptosis yielded 156 non-redundant phenotype combinations with OEadj>1 that were present in at least 3 probands for DRS or CFEOM or 2 probands for congenital ptosis (n=76, 58, and 22 for DRS, CFEOM, and congenital ptosis, respectively; Supplementary Table 4). Among these significant results were patterns of co-occurring phenotypes underlying a few recognizable syndromes. For instance, individuals with DRS and 10q deletion syndrome had CNS structural/functional malformation, PNS/muscle/connective tissue, skeletal (scoliosis), and renal/urinary/genital involvement (OEadj=6.04); individuals with Duane radial-ray syndrome had skeletal and renal involvement (OEadj=1.71); and individuals with CFEOM and ciliopathies had CNS structural/functional malformation, PNS/muscle/connective tissue, craniofacial, skeletal (non-scoliosis), and endocrine involvement (OEadj=9.05). While CODA analysis highlighted that many probands shared groupings of affected systems, their specific underlying phenotypes or genetic diagnoses often differed, so this analysis did not identify additional novel syndromes. We suggest that similar future analyses include more specific endophenotypes rather than broad systemic groupings to facilitate the identification of additional syndromes or to generate more informative pedigree groupings for analysis of shared genetic etiologies of syndromic phenotypes.

Brain MRI Findings in Sequenced Probands

To the extent feasible, MRI scans were assessed for cranial nerve and/or extraocular muscle anomalies, additional brain anomalies, and other non-brain anomalies (Supplementary Table 5, Fig. 2E). In total, 81 probands (83 affected individuals) had brain and/or orbital MRIs, of which scans from 47 probands (49 affected individuals) were available for review. Of these 47 probands, 25 had CFEOM (53.2%) and 11 had DRS (23.4%), while 4 had congenital ptosis, 3 had CN6-palsy, 3 had CCDD-NOS, and one had Brown syndrome (Supplementary Table 5, Fig. 2E). Thirty-one scans permitted interpretation of cranial nerve and/or extraocular muscle anatomy, of which 13 were optimized for cranial nerve/ extraocular muscle detection (8 CFEOM,

medRxiv preprint doi: https://doi.org/10.1101/2024.03.22.24304594; this version posted March 26, 2024. The copyright holder for this preprint (which was not certified by peer review) is the author/funder, who has granted medRxiv a license to display the preprint in perpetuity.
It is made available under a CC-BY 4.0 International license .

4 DRS, and 1 CCDD-NOS).

Findings in internally reviewed MRIs: Six of eight CFEOM probands with optimized MRIs had detectable thinning or absence of CN3; in five, CN3-innervated extraocular muscle(s) could also be assessed and were small. Exceptions included two CFEOM probands who, contrary to expectation, had normal-appearing CN3. One of these had normal CN3 but small CN3-innervated extraocular muscles (pedigree 41), suggesting possible limitations in detecting cranial nerve misrouting, defasciculation, or thinning. The second CFEOM proband with normal-appearing CN3 had orbital bands tethering the extraocular muscles (pedigree 99), suggesting a non-CCDD etiology as the cause of the clinical oCCDD diagnosis. All four DRS probands with optimized MRIs had thin/ absent CN6; while two had thin lateral rectus muscles, the lateral rectus muscles were normal-appearing in two, consistent with maintenance of the muscle secondary to aberrant innervation by CN3 in DRS (Supplementary Table 5).

Among probands with optimized MRIs, 9/13 (69.2%) had consistent laterality between their clinically detected oCCDD versus their MRI-derived cranial nerve/ extraocular muscle abnormalities. Inconsistencies were detected in 2 CFEOM probands and included clinically bilateral oCCDDs but unilateral MRI findings (pedigrees ENG_ASV and 260), again suggesting limitations in resolving subtle cranial nerve thinning. Interestingly, the only pedigree with MRI for >1 sequenced individual (pedigree 260 with CFEOM) had a mixed intrafamilial presentation with variable brain and other findings on MRI among different members (Supplementary Table 5).

Scans revealed diverse combinations of additional structural brain anomalies in 34/47 probands (72.3%). Commonly affected structures across all oCCDDs included the corpus callosum (17/34, 50.0%), cerebral ventricles (14, 41.2%), cerebellum (14, 41.2%), cerebral cortex (11, 32.4%), anterior commissure (11, 32.4%), and hippocampus (10, 29.4%) (Fig. 2E;

medRxiv preprint doi: https://doi.org/10.1101/2024.03.22.24304594; this version posted March 26, 2024. The copyright holder for this preprint (which was not certified by peer review) is the author/funder, who has granted medRxiv a license to display the preprint in perpetuity.
It is made available under a CC-BY 4.0 International license .

Supplementary Table 5). Cerebral cortex, pons, and olfactory system were also more frequently affected in DRS than in CFEOM, while anterior commissure and midbrain were more frequently affected in CFEOM than in DRS, but these associations were not statistically significant (chi-square test of independence, Fig. 2E). While some of these trends were expected (e.g. increased involvement of anterior commissure and midbrain in CFEOM), others were not (e.g. increased olfactory system involvement in DRS). This may be because imaged individuals often had atypical syndromic presentations, but may be in part because previous studies have not systematically ascertained these structures in all oCCDD subgroups.

Imaging revealed non-brain anomalies in 29/47 probands (61.7%). Common anomalies across all oCCDDs were in skull shape (9/29 31.0%), inner ear (7, 24.1%), vasculature (7, 24.1%), spine (6, 20.7%), internal auditory canals (4, 13.8%), and craniofacial structures (4, 13.8%) (Fig. 2E; Supplementary Table 5). While spine anomalies were more commonly observed in DRS than in CFEOM (3/7 versus 1/16; chi-square test of independence, X^2=4.54, df=1, p=0.033), this may have been due in part to targeted imaging of the spine in these individuals. Craniofacial and skull shape anomalies and microcephaly were more common in CFEOM, while inner ear anomalies were more common in DRS, but these associations were not statistically significant on evaluation by the chi-square test of independence.

Findings in externally reviewed MRIs: We received written reports but were unable to obtain MRIs for review from 34 probands. Seven were reported to have cranial nerve/ extraocular muscle findings (cranial nerve abnormalities in 3; extraocular muscle abnormalities in 5). Of these, imaging was reported to be optimized for cranial nerve/ extraocular muscle detection in 2, both of which showed consistency in laterality of the clinically detected oCCDD and the cranial nerve/ extraocular muscle findings. Among externally reported MRIs, 10 had structural brain abnormalities. Common findings were in the corpus callosum (3/10, 30.0%), white matter

medRxiv preprint doi: https://doi.org/10.1101/2024.03.22.24304594; this version posted March 26, 2024. The copyright holder for this preprint (which was not certified by peer review) is the author/funder, who has granted medRxiv a license to display the preprint in perpetuity.
It is made available under a CC-BY 4.0 International license .

volume (30.0%), cerebral cortex (20.0%), cerebellum (20.0%), pons (20.0%), and medulla (20.0%) (Supplementary Table 5).

Pre-existing genetic diagnoses

Clinical genetic findings identified before exome/genome sequencing explained the syndromic non-CCDD phenotypes in two individuals. These were proband ENG_AKL, who had congenital ptosis and mosaic Turner syndrome, and affected individual 178_04, who was not the proband of their pedigree but had DRS and Klinefelter syndrome (Supplementary Table 1).

Analysis of genes from SNV/indel analyses and their animal model phenotypes

The following 2000 genes had putatively relevant animal models in the Monarch database[28] under each mode of inheritance: XLR (6 genes), AR (57 genes), and AD (1973 genes); 36 genes were represented in both the AD and AR categories. Some model phenotypes were in generic neurodevelopmental processes, while others were more specifically oCCDD-related (detailed phenotypes provided in Supplementary Table 8).

The variants/genes in each pedigree that had compelling animal model data were then evaluated for full-gene and local missense constraint, predicted variant pathogenicity, population frequency, protein domain localization, relative location of pedigrees' variants, and biological function. Human probands with variants in the same gene were evaluated for phenotypic consistency with one another and with their animal model orthologs. This resulted in the prioritization of 95 variants in 59 genes among 89 pedigrees. Among these were candidate variants in genes without known human oCCDD involvement. The logic for prioritizing *NES, CUX1, GNAS, FER, ACTR1B, OLIG2,* and *SEMA3F* as putative novel candidate genes is provided below, while *KIF5C* is provided in the main text.

medRxiv preprint doi: https://doi.org/10.1101/2024.03.22.24304594; this version posted March 26, 2024. The copyright holder for this preprint (which was not certified by peer review) is the author/funder, who has granted medRxiv a license to display the preprint in perpetuity.
It is made available under a CC-BY 4.0 International license .

The intermediate filament protein-encoding gene *NES* harbored an AD variant in isolated familial CFEOM pedigree 251 that was rare and predicted damaging (c.23A>T, p.(Glu8Val), NM_006617.2; Supplementary Table 7). *NES* orthologous zebrafish mutants have multiple phenotypes, including small, disorganized, and apoptotic midbrain; multiple abnormal cranial nerves including CN3 and CN4; decreased neuronal precursors; and abnormal neuron differentiation (Supplementary Table 8).[33] *NES* is also highly expressed in adult human extraocular muscles, suggesting that alternative non-CCDD etiologies could also be involved. However, this gene has a compelling variant in just one pedigree in our cohort.[34]

CUX1, encoding a transcription factor involved in neuronal differentiation, is mutated in 3 DRS pedigrees: isolated sporadic DRS singletons ENG_PQ (c.724A>G, p.(Met242Val)) and ENG_GH (c.3853A>G, p.(Ile1285Val)) and isolated familial DRS trio 230 (c.3793G>A, p.(Glu1265Lys), NM_001202543.2) (Supplementary Table 7). All three variants are rare or absent from population databases and have moderate to damaging predictions. Residues 1265 and 1285 are in the protein homedomain, whereas residue 242 is not in an annotated domain. *CUX1* is highly missense-constrained within humans (missense z=3.749).[16] Notably, CUX1 binds to known DRS-associated protein CHN1,[35] and a mutant *CUX1* fly ortholog has abnormal neuroanatomy and neurophysiology (Supplementary Table 8). Largely LOF DNVs in *CUX1* have been reported in global developmental delays with or without intellectual disabilities (MIM116896), a phenotype which was not documented in any of our three pedigrees.

GNAS, encoding a G-protein whose signaling modulates hormones and neurotransmitters, is mutated in 4 DRS singletons: isolated sporadic DRS probands ENG_UE (c.713G>A, p.(Gly238Glu), NM_016592.5), ENG_JU (c.1591C>T, p.(Pro531Ser), NM_080425.4), and ENG_AAJ (c.1717G>C, p.(Asp573His), NM_080425.4) and syndromic sporadic DRS proband ENG_KS (c.304G>C, p.(Glu102Gln), NM_016592.5) (Supplementary Table 7). *GNAS* is

medRxiv preprint doi: https://doi.org/10.1101/2024.03.22.24304594; this version posted March 26, 2024. The copyright holder for this preprint (which was not certified by peer review) is the author/funder, who has granted medRxiv a license to display the preprint in perpetuity.
It is made available under a CC-BY 4.0 International license .

missense-constrained within humans (missense z= z-score: 2.655),[16] and the variants are rare and have moderate to damaging predictions. Interestingly, many of the syndromic features of proband ENG_KS have been previously associated with *GNAS* variants[36] (Supplementary Table 7), but DRS has not. Orthologous worm mutants have microtubule cytoskeleton abnormalities, and fly mutants have abnormal neurophysiology and smell perception (Supplementary Table 8).

FER is mutated in two singletons with isolated sporadic DRS (*FER*: ENG_1616, c.1883C>T, p.(Thr628Ile); ENG_1637, c.1887A>C, p.(Gln629His), NM_001308028.2; Supplementary Table 7). Both variants are absent from population databases and have moderate to damaging predictions. Interestingly, *FER* encodes a protein tyrosine kinase that regulates diverse processes including synaptic vesicle trafficking, actin cytoskeleton regulation, and microtubule assembly, and the *FER* variant residues in ENG_1616 and ENG_1637 are directly adjacent to one another in the protein kinase domain. *FER* worm models have abnormalities of axon midline crossing and receptor-mediated endocytosis (Supplementary Table 8).

ACTR1B is mutated in two singletons with isolated sporadic DRS (ENG_CMJ, c.1006C>G, p.(Arg336Gly); ENG_BAE, c.633T>A, p.(Phe211Leu), NM_005735.4; Supplementary Table 7). Both variants are absent from population databases and have moderate to damaging predictions. *ACTR1B* encodes a protein involved in vesicle movement along the microtubule, and worm models have defective receptor-mediated endocytosis (Supplementary Table 8).

OLIG2 encodes a transcription factor and is mutated in isolated familial DRS pedigree ENG_ET (c.467G>T, p.(Arg156Leu); Supplementary Table 7). The variant is absent from population databases and has damaging predictions, and *OLIG2* is moderately missense-constrained within humans. *Olig2* mouse models have fewer motor neurons and abnormalities of neuronal migration and the hindbrain, while fish models have abnormalities of neuronal migration, CN6,

medRxiv preprint doi: https://doi.org/10.1101/2024.03.22.24304594; this version posted March 26, 2024. The copyright holder for this preprint (which was not certified by peer review) is the author/funder, who has granted medRxiv a license to display the preprint in perpetuity.
It is made available under a CC-BY 4.0 International license .

and CN7. Additionally, worm models have abnormal neuronal cell fate specification and axon outgrowth (Supplementary Table 8).

Finally, *SEMA3F*, encoding a semaphorin involved in axon guidance, is mutated in syndromic sporadic CFEOM pedigree ENG_CMK (c.1889C>A, p.(Ser630Ter); Supplementary Table 7). The variant is absent from population databases and has damaging predictions, and *SEMA3F* is LOF-constrained within humans (LOEUF: 0.2190). *Sema3f*$^{-/-}$ mice have abnormalities of CN3 and CN4, neuronal migration, and axon guidance and fasciculation. Moreover, worm and fly models have abnormalities of axon guidance and of neuroanatomy and neurophysiology, respectively (Supplementary Table 8). In humans, heterozygous *SEMA3F* missense or LOF variants can result in hypogonadotropic hypogonadism,[37] which has not been documented in ENG_CMK.

DenovolyzeR analysis of *de novo* SNVs/indels

After filtering, we identified 297 DNVs (173 missense, 65 synonymous, 30 frameshift, 16 splice site,13 nonsense) among 200 probands. 232 genes had missense, frameshift, splice site, or nonsense variants; 9 genes were mutated twice, but 6 of these were mutated twice in single individuals. Twenty-three genes were not represented in denovolyzeR and were excluded from analysis.

Three genes each had missense DNVs in 2 probands (*TUBA1A, P2RX3*, and *SLC22A6*), but this enrichment did not meet statistical significance after Bonferroni correction for multiple gene testing (p=1.88e-5,1.82e-5, 2.75e-5 for the three genes, respectively; significance threshold at α=0.05 is 1.3e-6). The *TUBA1A* DNV in one of two probands is one of the variants we have reported as causal for the proband's syndromic CFEOM (pedigree 38; Individual 1[38]), while the second proband had syndromic DRS and has not been reported to date (pedigree 170;

medRxiv preprint doi: https://doi.org/10.1101/2024.03.22.24304594; this version posted March 26, 2024. The copyright holder for this preprint (which was not certified by peer review) is the author/funder, who has granted medRxiv a license to display the preprint in perpetuity.
It is made available under a CC-BY 4.0 International license .

c.1136G>A, p.(Ser379Asn), transcript NM_006009.4). While DRS has not been associated with *TUBA1A*, other aberrant innervation patterns have been, and our proband's syndromic findings are consistent with phenotypes reported in other individuals with *TUBA1A* variants. By contrast, the oCCDD in one of the probands with an *SLC22A6* DNV was solved by another genetic etiology and reported previously (pedigree 144 in our cohort; reported as pedigree 22[39]), and the two individuals with *P2RX3* DNVs have very disparate phenotypes (syndromic CN4-palsy and isolated DRS, respectively), making these less likely to be pathogenic.

Categorization of genes with ACMG/AMP/ClinGen-classifed SNVs/indels or SVs

The genes which harbored ACMG/AMP/ClinGen-classified P/LP or VUS SNVs/indels or SVs fell into five categories. Among the 14 probands with 14 variants in 7 [oCCDD+,Syndrome+/-] genes *KIF21A, TUBB3, PHOX2A, MAFB, CHN1, SALL4,* and *ROBO3*, five were not prescreened, five were prescreened but had VUS and were sequenced to exclude alternative causes, one was an SV that could not be detected with Sanger sequencing, one had one convincing *ROBO3* allele on pre-screening but no convincing second allele in this gene associated with a recessive condition, and two were missed on prescreening. Among these genes, *KIF21A, TUBB3*, and *ROBO3* had variants in both the P/LP and VUS categories.

The 40 [oCCDD(+),Syndrome+] genes that harbored 61 variants among 56 probands were *PIEZO2, KIAA0586* (compound heterozygous variants in a single proband)*, KIFBP* (compound heterozygous variants in a single proband)*, FGD1, PHOX2B, TRPV4, KMT2D, PTPN11* (mutated in 2 probands)*, ACTB, MED13* (mutated in 2 probands)*, EBF3* (mutated in 3 probands)*, ZC4H2* (mutated in 2 probands)*, BBS1* (compound heterozygous variants in a single proband)*, DMD, HDAC8, GCH1, DYRK1A, ZNF462* (mutated in 4 probands), *TGFBR2* (mutated in 2 probands)*, FBN1, POGZ, ARMC9* (mutated in 2 probands), *ECEL1, COL25A1, KIF21B, TUBB6* (mutated in 2 probands)*, CHD7, OPA1, TOGARAM1* (compound heterozygous variants

medRxiv preprint doi: https://doi.org/10.1101/2024.03.22.24304594; this version posted March 26, 2024. The copyright holder for this preprint (which was not certified by peer review) is the author/funder, who has granted medRxiv a license to display the preprint in perpetuity.
It is made available under a CC-BY 4.0 International license .

in a single proband), *WDR5, MCM3AP* (compound heterozygous variants in a single proband), *CDC42BPB, TUBB4A, OTUD6B* (compound heterozygous variants in a single proband), *FLNA, MPZ* (mutated in 2 probands), *ARX, WDR37, HNRNPK, MYH10* (mutated in 5 probands). Among these genes, *ARMC9*, *PTPN11, ZNF462,* and *TGFBR2* had variants in both the P/LP and VUS categories.

The 13 [oCCDD-,Syndrome+] genes that harbored 18 variants among 17 probands were *ARMC4, SCN1A, CDK13, FOXG1, TGFB2, GJB2, CEP83* (compound heterozygous variants in a single proband), *TUBA1A* (mutated in 2 probands), *HRAS, COL7A1, SLC12A5, GNAS* (mutated in 4 probands), *MACF1.* Among these genes, *CEP83* and *TUBA1A* had variants in both the P/LP and VUS categories.

The 16 [oCCDD-,Syndrome-] genes that harbored 24 VUS among 24 probands were *TUBA8, TUBA4A* (mutated in 3 probands), *SEMA3F, OLIG2, FRMD4B, TUBA3E, TUBA1B, TUBB, CTNNA1, KLB, FGF21, FER* (mutated in 2 probands), *ACTR1B* (mutated in 2 probands), *KIF5C* (mutated in 3 probands), *NES, CUX1* (mutated in 3 probands).

The 4 [Misdiagnoses] genes that harbored 5 variants among 5 probands were *FOXL2* (mutated in two probands), *RYR1, TWIST1*, *CHRNE*. Among these genes, *FOXL2* had a variant in both the P/LP and VUS categories.

Characteristics of oCCDD probands with ACMG/AMP/ClinGen-P/LP variants

As summarized in Supplementary Table 7, column K, the 13 pedigrees for which ACMG/AMP/ClinGen-P/LP SNV/indels or SVs fully explained the phenotypes are 198, ENG_AWA, ENG_ABW, 81, ENG_JP, 269, 270, 38, ENG_BS, 48, ENG_AKG, ENG_0640, and 193. The 13 pedigrees for which ACMG/AMP/ClinGen-P/LP variant(s) explained the syndromic

medRxiv preprint doi: https://doi.org/10.1101/2024.03.22.24304594; this version posted March 26, 2024. The copyright holder for this preprint (which was not certified by peer review) is the author/funder, who has granted medRxiv a license to display the preprint in perpetuity.
It is made available under a CC-BY 4.0 International license .

but not the oCCDD phenotype were 239, ENG_1894, 242, 93, 128, ENG_2270, 42, 227, 238, ENG_AHO, ENG_ADU, ENG_ACR, 257. The 3 pedigrees in which the P/LP variants explained the oCCDD but not the syndromic phenotype were 27, 13, ENG_CMO. The 14 pedigrees in which both the oCCDD and non-CCDD phenotypes were expanded are ENG_BAG, ENG_AGZ, 4, ENG_CKA, ENG_CHA, 61, ENG_FI, 144, 71, 233, 131, 26, 62, ENG_AZW. In two cases, a P/LP allele was compound heterozygous with a VUS in a gene that fully or partially explained the phenotype (pedigrees 193 and ENG_AZW, respectively).

Some VUS had higher levels of supportive evidence and compatibility with prior reported oCCDD and/or syndromic phenotypes consistent with the phenotypes of the probands who harbored them, suggesting a higher likelihood of their being substantiated over time. These included 14 variants in 13 genes among 14 pedigrees: *SALL4* (ENG_DQ), *CHN1* (ENG_1580, ENG_BBG), *MAFB* (232), *PHOX2A* (160), *TUBA1A* (170), *ECEL1* (223), *COL25A1* (56),[40] *MACF1* (98),[41] *ARMC9* (ENG_COX), *KIF21B* (ENG_FR), *TUBB6* (ENG_CML), *TUBA4A* (ENG_IM), and *CHRNE* (ENG_2044) (Table 1, Fig. 3H, Supplementary Table 7).

Among oCCDD subphenotypes, ACMG/AMP/ClinGen-P/LP variants were obtained in the following numbers of probands: horizontal gaze palsy=3/6 (50.0%), CCDD-NOS=4/9 (44.4%), CN6-palsy=1/5 (20.0%), CFEOM=14/80 (17.5%), Brown syndrome=2/13 (15.4%), congenital ptosis=7/75 (9.3%), DRS=10/198 (5.1%), CN4-palsy=1/28 (3.6%), and MGJWS=1/53 (1.9%). Additional breakdowns are provided (Fig. 3I, Supplementary Figure 10).

medRxiv preprint doi: https://doi.org/10.1101/2024.03.22.24304594; this version posted March 26, 2024. The copyright holder for this preprint (which was not certified by peer review) is the author/funder, who has granted medRxiv a license to display the preprint in perpetuity.
It is made available under a CC-BY 4.0 International license .

SUPPLEMENTARY REFERENCES

1. World Health Organization. *Birth Defects Surveillance: A Manual for Programme Managers*.; 2015. https://books.google.com/books/about/Birth_Defects_Surveillance.html?hl=&id=eIGErgEACAAJ

2. Köhler S, Gargano M, Matentzoglu N, et al. The Human Phenotype Ontology in 2021. *Nucleic Acids Res*. 2021;49(D1):D1207-D1217. doi:10.1093/nar/gkaa1043

3. Benjamin RH, Yu X, Navarro Sanchez ML, et al. Co-occurring defect analysis: A platform for analyzing birth defect co-occurrence in registries. *Birth Defects Res*. 2019;111(18):1356-1364. doi:10.1002/bdr2.1549

4. Schneider VA, Graves-Lindsay T, Howe K, et al. Evaluation of GRCh38 and de novo haploid genome assemblies demonstrates the enduring quality of the reference assembly. *Genome Res*. 2017;27(5):849-864. doi:10.1101/gr.213611.116

5. Li H, Durbin R. Fast and accurate short read alignment with Burrows-Wheeler transform. *Bioinformatics*. 2009;25(14):1754-1760. doi:10.1093/bioinformatics/btp324

6. McKenna A, Hanna M, Banks E, et al. The Genome Analysis Toolkit: a MapReduce framework for analyzing next-generation DNA sequencing data. *Genome Res*. 2010;20(9):1297-1303. doi:10.1101/gr.107524.110

7. DePristo MA, Banks E, Poplin R, et al. A framework for variation discovery and genotyping using next-generation DNA sequencing data. *Nat Genet*. 2011;43(5):491-498. doi:10.1038/ng.806

8. McLaren W, Gil L, Hunt SE, et al. The Ensembl Variant Effect Predictor. *Genome Biol*. 2016;17(1):122. doi:10.1186/s13059-016-0974-4

9. Pais LS, Snow H, Weisburd B, et al. seqr: A web-based analysis and collaboration tool for rare disease genomics. *Hum Mutat*. 2022;43(6):698-707. doi:10.1002/humu.24366

10. Karczewski KJ, Francioli LC, Tiao G, et al. The mutational constraint spectrum quantified from variation in 141,456 humans. *Nature*. 2020;581(7809):434-443. doi:10.1038/s41586-020-2308-7

11. 1000 Genomes Project Consortium, Auton A, Brooks LD, et al. A global reference for human genetic variation. *Nature*. 2015;526(7571):68-74. doi:10.1038/nature15393

12. Lek M, Karczewski KJ, Minikel EV, et al. Analysis of protein-coding genetic variation in 60,706 humans. *Nature*. 2016;536(7616):285-291. doi:10.1038/nature19057

13. Taliun D, Harris DN, Kessler MD, et al. Sequencing of 53,831 diverse genomes from the NHLBI TOPMed Program. *Nature*. 2021;590(7845):290-299. doi:10.1038/s41586-021-03205-y

14. Freeman PJ, Hart RK, Gretton LJ, Brookes AJ, Dalgleish R. VariantValidator: Accurate validation, mapping, and formatting of sequence variation descriptions. *Hum Mutat*. 2018;39(1):61-68. doi:10.1002/humu.23348

medRxiv preprint doi: https://doi.org/10.1101/2024.03.22.24304594; this version posted March 26, 2024. The copyright holder for this preprint (which was not certified by peer review) is the author/funder, who has granted medRxiv a license to display the preprint in perpetuity.
It is made available under a CC-BY 4.0 International license .

15. Rentzsch P, Witten D, Cooper GM, Shendure J, Kircher M. CADD: predicting the deleteriousness of variants throughout the human genome. *Nucleic Acids Res*. 2019;47(D1):D886-D894. doi:10.1093/nar/gky1016

16. Samocha KE, Robinson EB, Sanders SJ, et al. A framework for the interpretation of de novo mutation in human disease. *Nat Genet*. 2014;46(9):944-950. doi:10.1038/ng.3050

17. Hamosh A, Amberger JS, Bocchini C, Scott AF, Rasmussen SA. Online Mendelian Inheritance in Man (OMIM®): Victor McKusick's magnum opus. *Am J Med Genet A*. 2021;185(11):3259-3265. doi:10.1002/ajmg.a.62407

18. Stenson PD, Mort M, Ball EV, et al. The Human Gene Mutation Database: towards a comprehensive repository of inherited mutation data for medical research, genetic diagnosis and next-generation sequencing studies. *Hum Genet*. 2017;136(6):665-677. doi:10.1007/s00439-017-1779-6

19. Landrum MJ, Lee JM, Benson M, et al. ClinVar: improving access to variant interpretations and supporting evidence. *Nucleic Acids Res*. 2018;46(D1):D1062-D1067. doi:10.1093/nar/gkx1153

20. Ware JS, Samocha KE, Homsy J, Daly MJ. Interpreting de novo Variation in Human Disease Using denovolyzeR. *Curr Protoc Hum Genet*. 2015;87:7.25.1-7.25.15. doi:10.1002/0471142905.hg0725s87

21. Kolberg L, Raudvere U, Kuzmin I, Adler P, Vilo J, Peterson H. g:Profiler-interoperable web service for functional enrichment analysis and gene identifier mapping (2023 update). *Nucleic Acids Res*. 2023;51(W1):W207-W212. doi:10.1093/nar/gkad347

22. Collins RL, Brand H, Karczewski KJ, et al. A structural variation reference for medical and population genetics. *Nature*. 2020;581(7809):444-451. doi:10.1038/s41586-020-2287-8

23. Belyeu JR, Brand H, Wang H, et al. De novo structural mutation rates and gamete-of-origin biases revealed through genome sequencing of 2,396 families. *Am J Hum Genet*. 2021;108(4):597-607. doi:10.1016/j.ajhg.2021.02.012

24. Robinson JT, Thorvaldsdóttir H, Winckler W, et al. Integrative genomics viewer. *Nat Biotechnol*. 2011;29(1):24-26. doi:10.1038/nbt.1754

25. Collins RL, Glessner JT, Porcu E, et al. A cross-disorder dosage sensitivity map of the human genome. *Cell*. 2022;185(16):3041-3055.e25. doi:10.1016/j.cell.2022.06.036

26. Babadi M, Fu JM, Lee SK, et al. GATK-gCNV enables the discovery of rare copy number variants from exome sequencing data. *Nat Genet*. 2023;55(9):1589-1597. doi:10.1038/s41588-023-01449-0

27. Fu JM, Satterstrom FK, Peng M, et al. Rare coding variation provides insight into the genetic architecture and phenotypic context of autism. *Nat Genet*. 2022;54(9):1320-1331. doi:10.1038/s41588-022-01104-0

28. McMurry JA, Köhler S, Washington NL, et al. Navigating the Phenotype Frontier: The Monarch Initiative. *Genetics*. 2016;203(4):1491-1495. doi:10.1534/genetics.116.188870

29. Richards S, Aziz N, Bale S, et al. Standards and guidelines for the interpretation of

medRxiv preprint doi: https://doi.org/10.1101/2024.03.22.24304594; this version posted March 26, 2024. The copyright holder for this preprint (which was not certified by peer review) is the author/funder, who has granted medRxiv a license to display the preprint in perpetuity.
It is made available under a CC-BY 4.0 International license .

sequence variants: a joint consensus recommendation of the American College of Medical Genetics and Genomics and the Association for Molecular Pathology. *Genet Med*. 2015;17(5):405-424. doi:10.1038/gim.2015.30

30. Ioannidis NM, Rothstein JH, Pejaver V, et al. REVEL: An Ensemble Method for Predicting the Pathogenicity of Rare Missense Variants. *Am J Hum Genet*. 2016;99(4):877-885. doi:10.1016/j.ajhg.2016.08.016

31. Riggs ER, Andersen EF, Cherry AM, et al. Technical standards for the interpretation and reporting of constitutional copy-number variants: a joint consensus recommendation of the American College of Medical Genetics and Genomics (ACMG) and the Clinical Genome Resource (ClinGen). *Genet Med*. 2020;22(2):245-257. doi:10.1038/s41436-019-0686-8

32. Cheng J, Novati G, Pan J, et al. Accurate proteome-wide missense variant effect prediction with AlphaMissense. *Science*. 2023;381(6664):eadg7492. doi:10.1126/science.adg7492

33. Chen HL, Yuh CH, Wu KK. Nestin is essential for zebrafish brain and eye development through control of progenitor cell apoptosis. *PLoS One*. 2010;5(2):e9318. doi:10.1371/journal.pone.0009318

34. Janbaz AH, Lindström M, Liu JX, Pedrosa Domellöf F. Intermediate filaments in the human extraocular muscles. *Invest Ophthalmol Vis Sci*. 2014;55(8):5151-5159. doi:10.1167/iovs.14-14316

35. Schilders KAA, Edel GG, Eenjes E, et al. Identification of SOX2 Interacting Proteins in the Developing Mouse Lung With Potential Implications for Congenital Diaphragmatic Hernia. *Front Pediatr*. 2022;10:881287. doi:10.3389/fped.2022.881287

36. Haldeman-Englert CR, Hurst ACE, Levine MA. Disorders of Inactivation. In: Adam MP, Feldman J, Mirzaa GM, et al., eds. *GeneReviews*. University of Washington, Seattle; 2017. https://www.ncbi.nlm.nih.gov/pubmed/29072892

37. Kotan LD, Ternier G, Cakir AD, et al. Loss-of-function variants in SEMA3F and PLXNA3 encoding semaphorin-3F and its receptor plexin-A3 respectively cause idiopathic hypogonadotropic hypogonadism. *Genet Med*. 2021;23(6):1008-1016. doi:10.1038/s41436-020-01087-5

38. Jurgens JA, Barry BJ, Lemire G, et al. Novel variants in TUBA1A cause congenital fibrosis of the extraocular muscles with or without malformations of cortical brain development. *Eur J Hum Genet*. 2021;29(5):816-826. doi:10.1038/s41431-020-00804-7

39. Frints SGM, Hennig F, Colombo R, et al. Deleterious de novo variants of X-linked ZC4H2 in females cause a variable phenotype with neurogenic arthrogryposis multiplex congenita. *Hum Mutat*. 2019;40(12):2270-2285. doi:10.1002/humu.23841

40. Natera-de Benito D, Jurgens JA, Yeung A, et al. Recessive variants in COL25A1 gene as novel cause of arthrogryposis multiplex congenita with ocular congenital cranial dysinnervation disorder. *Hum Mutat*. 2022;43(4):487-498. doi:10.1002/humu.24333

41. Dobyns WB, Aldinger KA, Ishak GE, et al. MACF1 Mutations Encoding Highly Conserved Zinc-Binding Residues of the GAR Domain Cause Defects in Neuronal Migration and Axon Guidance. *Am J Hum Genet*. 2018;103(6):1009-1021. doi:10.1016/j.ajhg.2018.10.019

medRxiv preprint doi: https://doi.org/10.1101/2024.03.22.24304594; this version posted March 26, 2024. The copyright holder for this preprint (which was not certified by peer review) is the author/funder, who has granted medRxiv a license to display the preprint in perpetuity.
It is made available under a CC-BY 4.0 International license .

SUPPLEMENTARY WEB RESOURCES

http://broadinstitute.github.io/picard/

https://gatk.broadinstitute.org/hc/en-us

https://seqr.broadinstitute.org/

https://github.com/broadinstitute/tgg_methods/

Online Mendelian Inheritance in Man, OMIM®. McKusick-Nathans Institute of Genetic Medicine, Johns Hopkins University (Baltimore, MD). World Wide Web URL: https://omim.org/. Accessed May 2023.

Stenson et al (2003), The Human Gene Mutation Database (HGMD®): 2003 Update. Hum Mutat (2003) 21:577-581. HGMD Professional version 2021.4.

http://monarchinitiative.org

https://github.com/broadinstitute/gatk-sv

medRxiv preprint doi: https://doi.org/10.1101/2024.03.22.24304594; this version posted March 26, 2024. The copyright holder for this preprint (which was not certified by peer review) is the author/funder, who has granted medRxiv a license to display the preprint in perpetuity.
It is made available under a CC-BY 4.0 International license .

SUPPLEMENTARY FIGURES

Supplementary Figure 1. Human cranial nerve schematics

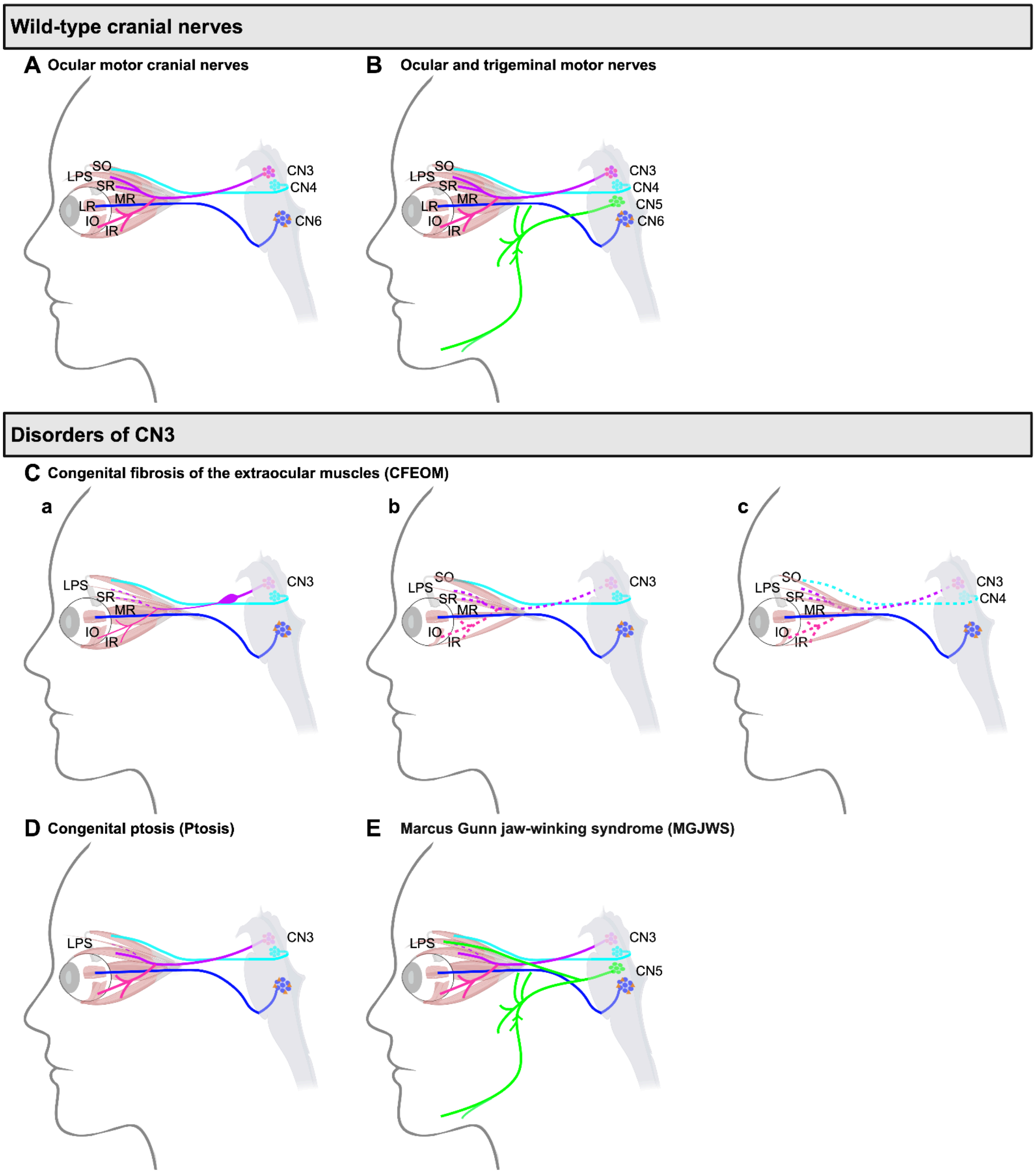

medRxiv preprint doi: https://doi.org/10.1101/2024.03.22.24304594; this version posted March 26, 2024. The copyright holder for this preprint (which was not certified by peer review) is the author/funder, who has granted medRxiv a license to display the preprint in perpetuity.
It is made available under a CC-BY 4.0 International license .

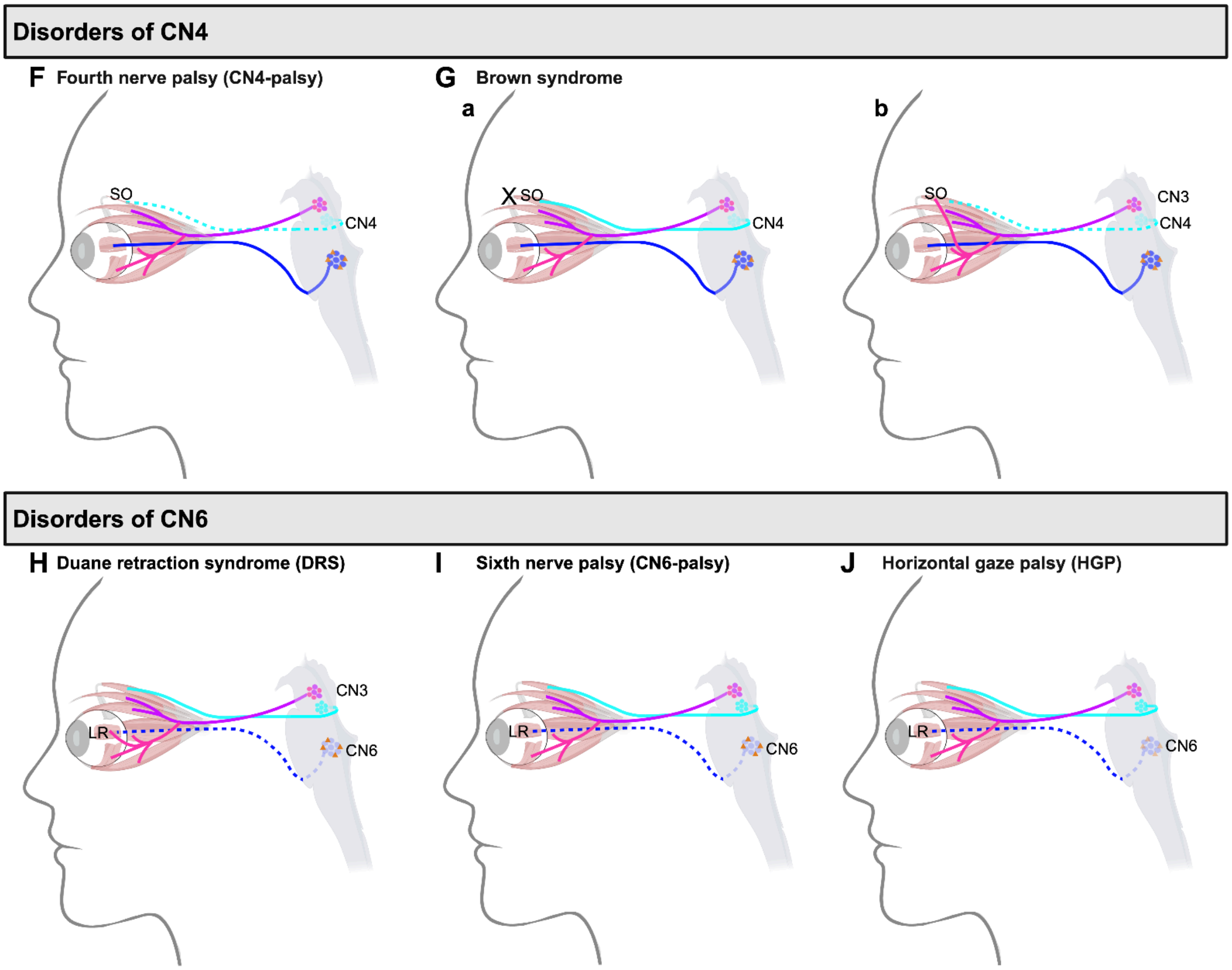

A: Wild-type human ocular motor cranial nerves. Three ocular motor cranial motor nuclei (clusters of cells in the brainstem; CN3, CN4, and CN6) give rise to cranial nerves (CN) that innervate 7 target muscles that control eye and/or eyelid movement (IO, IR, LPS, LR, MR, SO, SR). Errors in the identity or migration of these motor neurons or in their axonal growth or guidance can result in oCCDDs. CN3 originates from the midbrain, exits ventrally, and gives rise to a nerve with two main branches: a superior division (purple), which innervates the SR and LPS muscles, and an inferior division (magenta), which innervates the IO, IR, and MR muscles. The CN4 nucleus (cyan) in the midbrain gives rise to the CN4 nerve, which exits the midbrain dorsally, crosses the midline, and passes around the brainstem to innervate the contralateral SO muscle. The CN6 nucleus in the pons contains motor neurons (dark blue) whose axons exit ventrally and innervate the LR muscle, and interneurons (orange) that cross the midline and ascend to contact the medial rectus motor neurons in CN3. **B:** Wild-type human ocular motor cranial nerves are shown as in A, with the addition of the wild-type trigeminal motor nucleus and nerve (CN5), which innervates the muscles of mastication. **C-E:** CN3-related disorders include CFEOM, congenital ptosis, and MGJWS. **C:** CFEOM results from malformation of the superior division of CN3 with corresponding hypoplasia of the SR and LPS muscles (a-c) and can be characterized in mouse models by initial dilation followed by thinning of the nerve (a). CN3 superior division defects may be accompanied by malformation of the inferior division of CN3 (b) with IO, IR, and MR muscle hypoplasia, and, in some cases, malformation of CN4 and the SO muscle (c), or CN6 and the LR muscle (not shown). **D:** Congenital ptosis can be caused by maldevelopment of the superior branch of CN3 to the LPS with corresponding LPS hypoplasia. **E:** In congenital ptosis accompanied by MGJWS, the superior branch of CN3 to the LPS is deficient as in congenital ptosis, but the LPS is aberrantly innervated by CN5. The mechanisms and precise branches of CN5 involved in this phenotype are poorly understood. **F-G:** CN4-related disorders include CN4-palsy and Brown syndrome. **F:** In CN4-palsy, CN4 and its SO target muscle are hypoplastic. **G:** Brown syndrome can result from limited motility of the SO muscle or its tendon sheath (a). Some cases of Brown syndrome are alternatively hypothesized to be oCCDDs resulting from CN4-palsy with aberrant innervation, potentially from the inferior division of CN3 (b), but such mechanisms are not proven. **H-J:** CN6-related disorders include DRS, CN6-palsy, and HGP. **H:** DRS is characterized by CN6 motor neuron maldevelopment or axon stalling, with secondary aberrant innervation of the LR muscle by the inferior division of CN3. In DRS, the CN6 motor nucleus interneurons (orange) are spared. **I:** Congenital CN6-palsy arises secondary to CN6 maldevelopment or degeneration, with sparing of the interneurons. **J:** HGP can be caused by abnormalities of CN6, including its interneurons, or the brain regions that project to CN6, such as the medial longitudinal fasciculus or paramedian pontine reticular formation (not shown). Abbreviations: CFEOM=congenital fibrosis of the extraocular muscles, CN=cranial nerve, CN3=cranial nerve 3 (oculomotor), CN4=cranial nerve 4 (trochlear), CN4-palsy=fourth nerve palsy, CN5=cranial nerve 5 (trigeminal motor), CN6=cranial nerve 6 (abducens), CN6-palsy=sixth nerve palsy, DRS=Duane retraction syndrome, HGP=horizontal gaze palsy, IO=inferior oblique muscle, IR=inferior rectus muscle, LPS=levator palpebrae superioris muscle, LR=lateral rectus muscle, MGJWS=Marcus Gunn jaw-winking syndrome, MR=medial rectus muscle, SO=superior oblique muscle, SR=superior rectus muscle. Key: purple=CN3 superior division, pink=CN3 inferior division, cyan=CN4, green=CN5, dark blue=CN6, orange=CN6 interneurons, “X” or increased transparency=structure/function compromised, dashed nerve=nerve missing or hypoplastic.

medRxiv preprint doi: https://doi.org/10.1101/2024.03.22.24304594; this version posted March 26, 2024. The copyright holder for this preprint (which was not certified by peer review) is the author/funder, who has granted medRxiv a license to display the preprint in perpetuity.
It is made available under a CC-BY 4.0 International license .

Supplementary Figure 2. Sequencing type among 467 probands

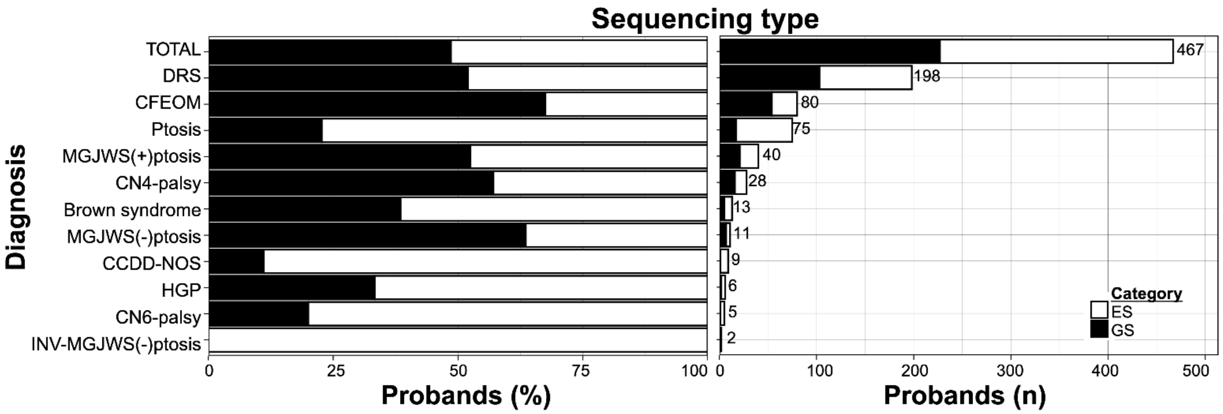

Abbreviations: CCDD=congenital cranial dysinnervation disorder, CCDD-NOS=CCDD not otherwise specified, CFEOM=congenital fibrosis of the extraocular muscles, CN4-palsy=fourth nerve palsy, CN6-palsy=sixth nerve palsy, DRS=Duane retraction syndrome, ES=exome sequencing, GS=genome sequencing, HGP=horizontal gaze palsy, INV-MGJWS(-)ptosis=inverse Marcus Gunn jaw-winking synkinesis without congenital ptosis, MGJWS(+)ptosis=Marcus Gunn jaw-winking synkinesis with congenital ptosis, MGJWS(-)ptosis=Marcus Gunn jaw-winking synkinesis without congenital ptosis, oCCDD=ocular congenital cranial dysinnervation disorder, Ptosis=congenital ptosis.

Supplementary Figure 3. Sequencing structure among 467 probands

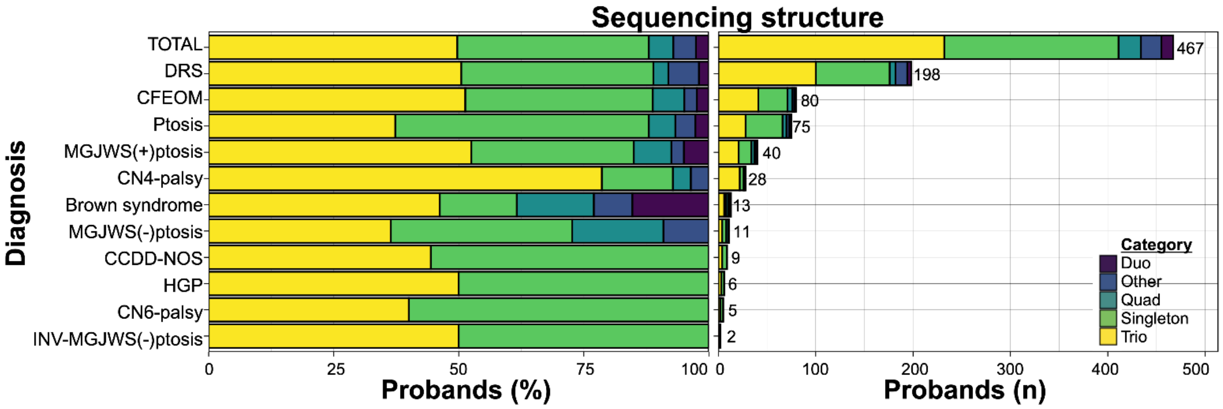

Abbreviations: CCDD=congenital cranial dysinnervation disorder, CCDD-NOS=CCDD not otherwise specified, CFEOM=congenital fibrosis of the extraocular muscles, CN4-palsy=fourth nerve palsy, CN6-palsy=sixth nerve palsy, DRS=Duane retraction syndrome, Duo=2 members of pedigree sequenced, HGP=horizontal gaze palsy, INV-MGJWS(-)ptosis=inverse Marcus Gunn jaw-winking synkinesis without congenital ptosis, MGJWS(+)ptosis=Marcus Gunn jaw-winking synkinesis with congenital ptosis, MGJWS(-)ptosis=Marcus Gunn jaw-winking synkinesis without congenital ptosis, oCCDD=ocular congenital cranial dysinnervation disorder, Other= more than 4 members of pedigree sequenced, Ptosis=congenital ptosis, Quad=4 members of pedigree sequenced, Singleton=1 member of pedigree sequenced, Trio=3 members of pedigree sequenced.

medRxiv preprint doi: https://doi.org/10.1101/2024.03.22.24304594; this version posted March 26, 2024. The copyright holder for this preprint (which was not certified by peer review) is the author/funder, who has granted medRxiv a license to display the preprint in perpetuity.
It is made available under a CC-BY 4.0 International license .

Supplementary Figure 4. Sporadic versus familial oCCDDs among 467 probands

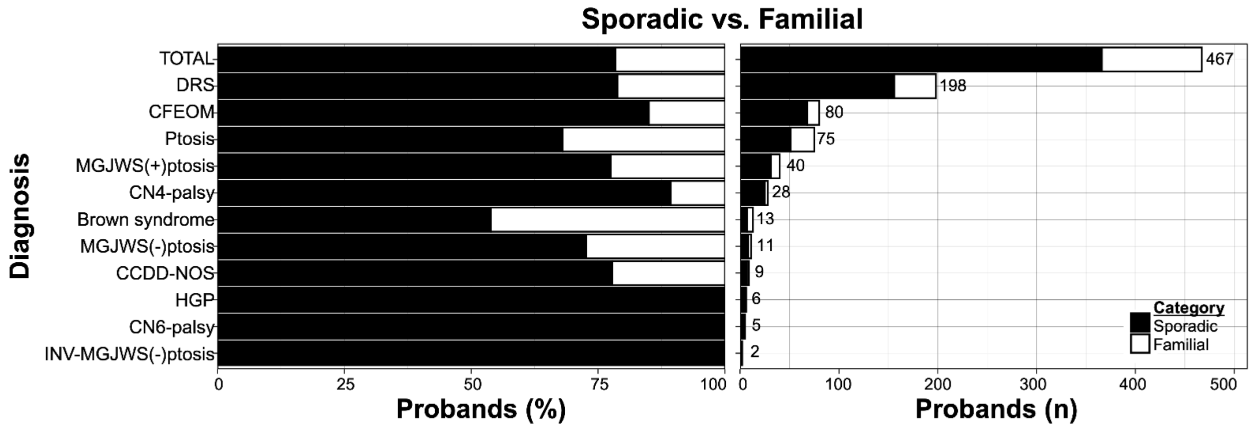

Abbreviations: CCDD=congenital cranial dysinnervation disorder, CCDD-NOS=CCDD not otherwise specified, CFEOM=congenital fibrosis of the extraocular muscles, CN4-palsy=fourth nerve palsy, CN6-palsy=sixth nerve palsy, DRS=Duane retraction syndrome, HGP=horizontal gaze palsy, INV-MGJWS(-)ptosis=inverse Marcus Gunn jaw-winking synkinesis without congenital ptosis, MGJWS(+)ptosis=Marcus Gunn jaw-winking synkinesis with congenital ptosis, MGJWS(-)ptosis=Marcus Gunn jaw-winking synkinesis without congenital ptosis, oCCDD=ocular congenital cranial dysinnervation disorder, Ptosis=congenital ptosis.

Supplementary Figure 5. Syndromic versus isolated oCCDD breakdown among 467 probands

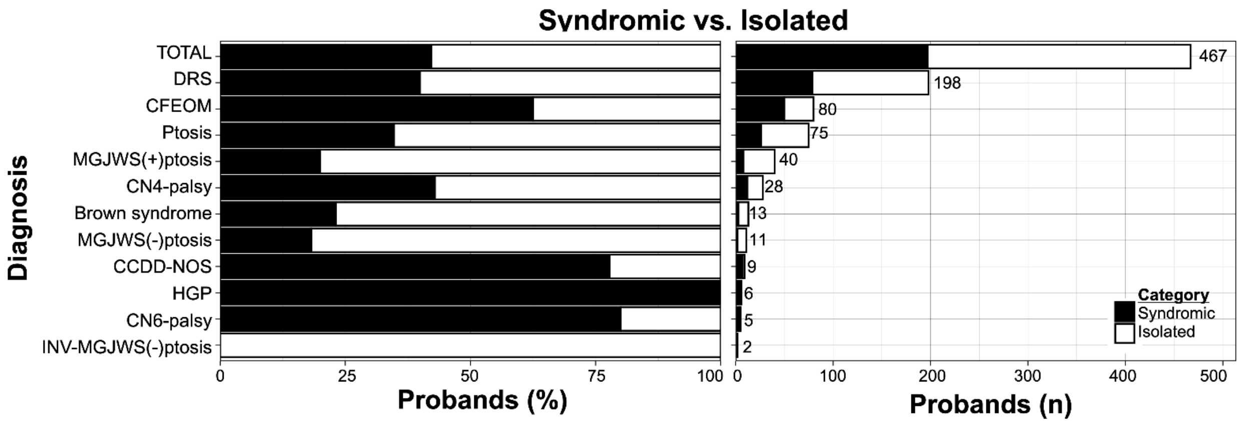

Abbreviations: CCDD=congenital cranial dysinnervation disorder, CCDD-NOS=CCDD not otherwise specified, CFEOM=congenital fibrosis of the extraocular muscles, CN4-palsy=fourth nerve palsy, CN6-palsy=sixth nerve palsy, DRS=Duane retraction syndrome, HGP=horizontal gaze palsy, INV-MGJWS(-)ptosis=inverse Marcus Gunn jaw-winking synkinesis without congenital ptosis, MGJWS(+)ptosis=Marcus Gunn jaw-winking synkinesis with congenital ptosis, MGJWS(-)ptosis=Marcus Gunn jaw-winking synkinesis without congenital ptosis, oCCDD=ocular congenital cranial dysinnervation disorder, Ptosis=congenital ptosis.

medRxiv preprint doi: https://doi.org/10.1101/2024.03.22.24304594; this version posted March 26, 2024. The copyright holder for this preprint
(which was not certified by peer review) is the author/funder, who has granted medRxiv a license to display the preprint in perpetuity.
It is made available under a CC-BY 4.0 International license .

Supplementary Figure 6. Combined sporadic/familial and isolated/syndromic oCCDD breakdown among 467 probands

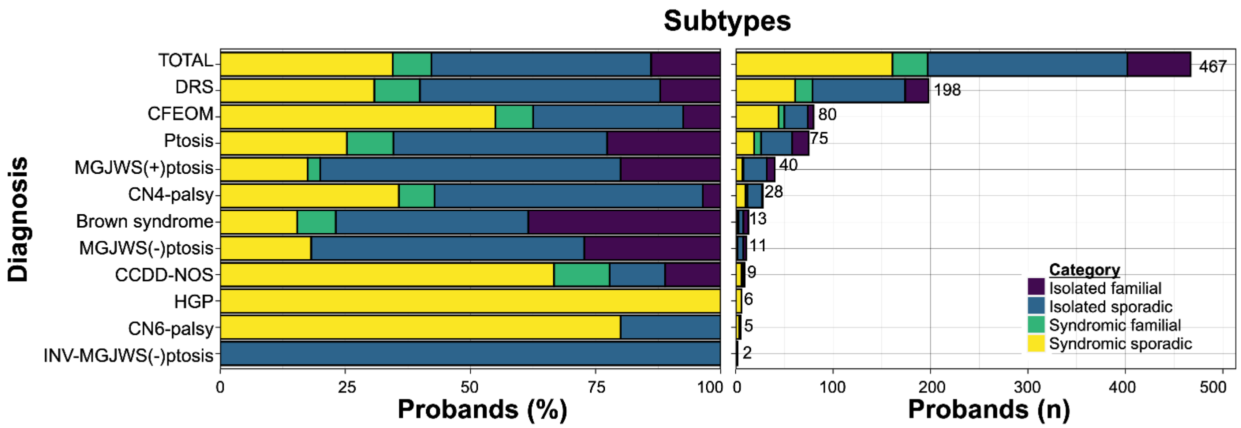

Abbreviations: CCDD=congenital cranial dysinnervation disorder, CCDD-NOS=CCDD not otherwise specified, CFEOM=congenital fibrosis of the extraocular muscles, CN4-palsy=fourth nerve palsy, CN6-palsy=sixth nerve palsy, DRS=Duane retraction syndrome, HGP=horizontal gaze palsy, INV-MGJWS(-)ptosis=inverse Marcus Gunn jaw-winking synkinesis without congenital ptosis, MGJWS(+)ptosis=Marcus Gunn jaw-winking synkinesis with congenital ptosis, MGJWS(-)ptosis=Marcus Gunn jaw-winking synkinesis without congenital ptosis, oCCDD=ocular congenital cranial dysinnervation disorder, Ptosis=congenital ptosis.

Supplementary Figure 7. Laterality of oCCDDs among 467 probands

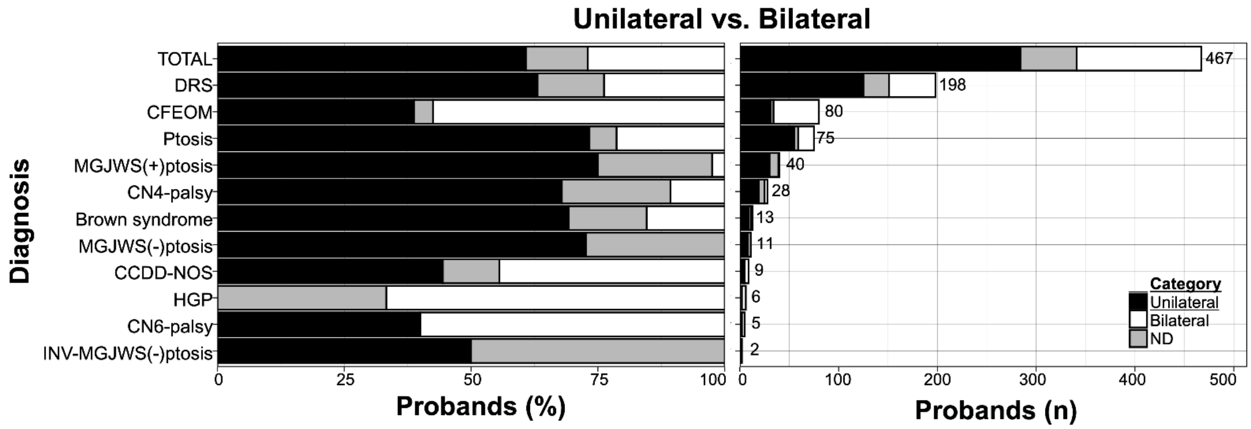

Abbreviations: CCDD=congenital cranial dysinnervation disorder, CCDD-NOS=CCDD not otherwise specified, CFEOM=congenital fibrosis of the extraocular muscles, CN4-palsy=fourth nerve palsy, CN6-palsy=sixth nerve palsy, DRS=Duane retraction syndrome, HGP=horizontal gaze palsy, INV-MGJWS(-)ptosis=inverse Marcus Gunn jaw-winking synkinesis without congenital ptosis, MGJWS(+)ptosis=Marcus Gunn jaw-winking synkinesis with congenital ptosis, MGJWS(-)ptosis=Marcus Gunn jaw-winking synkinesis without congenital ptosis, ND=not described, oCCDD=ocular congenital cranial dysinnervation disorder, Ptosis=congenital ptosis.

medRxiv preprint doi: https://doi.org/10.1101/2024.03.22.24304594; this version posted March 26, 2024. The copyright holder for this preprint (which was not certified by peer review) is the author/funder, who has granted medRxiv a license to display the preprint in perpetuity.
It is made available under a CC-BY 4.0 International license .

Supplementary Figure 8. Documented synkinesis among 467 probands

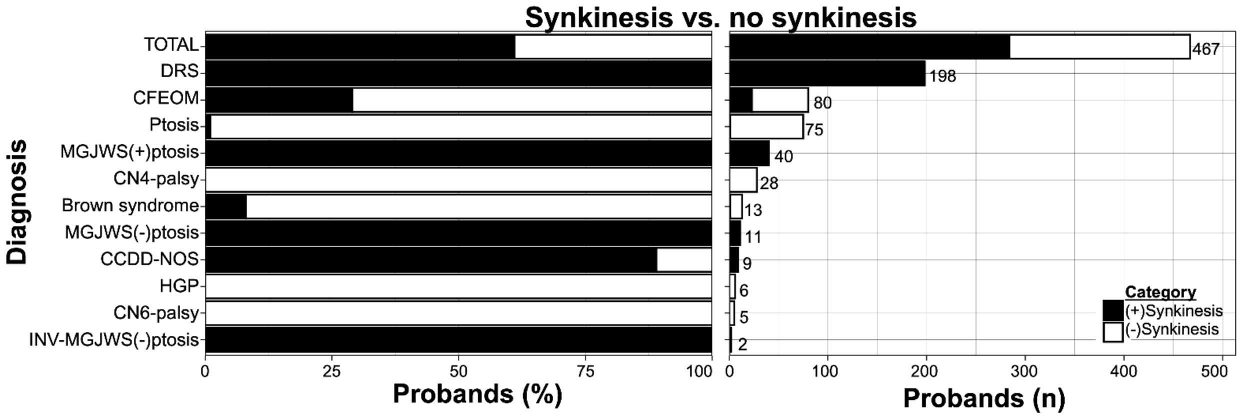

Abbreviations: CCDD=congenital cranial dysinnervation disorder, CCDD-NOS=CCDD not otherwise specified, CFEOM=congenital fibrosis of the extraocular muscles, CN4-palsy=fourth nerve palsy, CN6-palsy=sixth nerve palsy, DRS=Duane retraction syndrome, HGP=horizontal gaze palsy, INV-MGJWS(-)ptosis=inverse Marcus Gunn jaw-winking synkinesis without congenital ptosis, MGJWS(+)ptosis=Marcus Gunn jaw-winking synkinesis with congenital ptosis, MGJWS(-)ptosis=Marcus Gunn jaw-winking synkinesis without congenital ptosis, ND=not described, oCCDD=ocular congenital cranial dysinnervation disorder, Ptosis=congenital ptosis.

Supplementary Figure 9. Protein mapping of *MYH10* variants identified in our cohort and reported in the literature

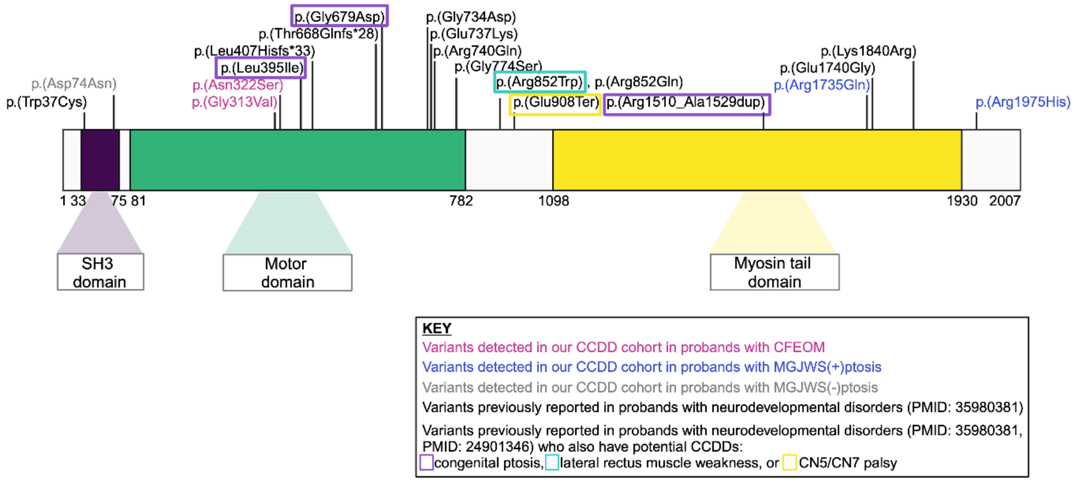

Abbreviations: CCDD=congenital cranial dysinnervation disorder, CFEOM=congenital fibrosis of the extraocular muscles, MGJWS(+)ptosis=Marcus Gunn jaw-winking synkinesis with congenital ptosis, MGJWS(-)ptosis=Marcus Gunn jaw-winking synkinesis without congenital ptosis, Ptosis=congenital ptosis. Variants mapped with ENST00000360416.8 (NM_001256012.3).

medRxiv preprint doi: https://doi.org/10.1101/2024.03.22.24304594; this version posted March 26, 2024. The copyright holder for this preprint
(which was not certified by peer review) is the author/funder, who has granted medRxiv a license to display the preprint in perpetuity.
It is made available under a CC-BY 4.0 International license .

Supplementary Figure 10. Characteristics among pedigrees with ACMG/AMP/ClinGen-P/LP variants

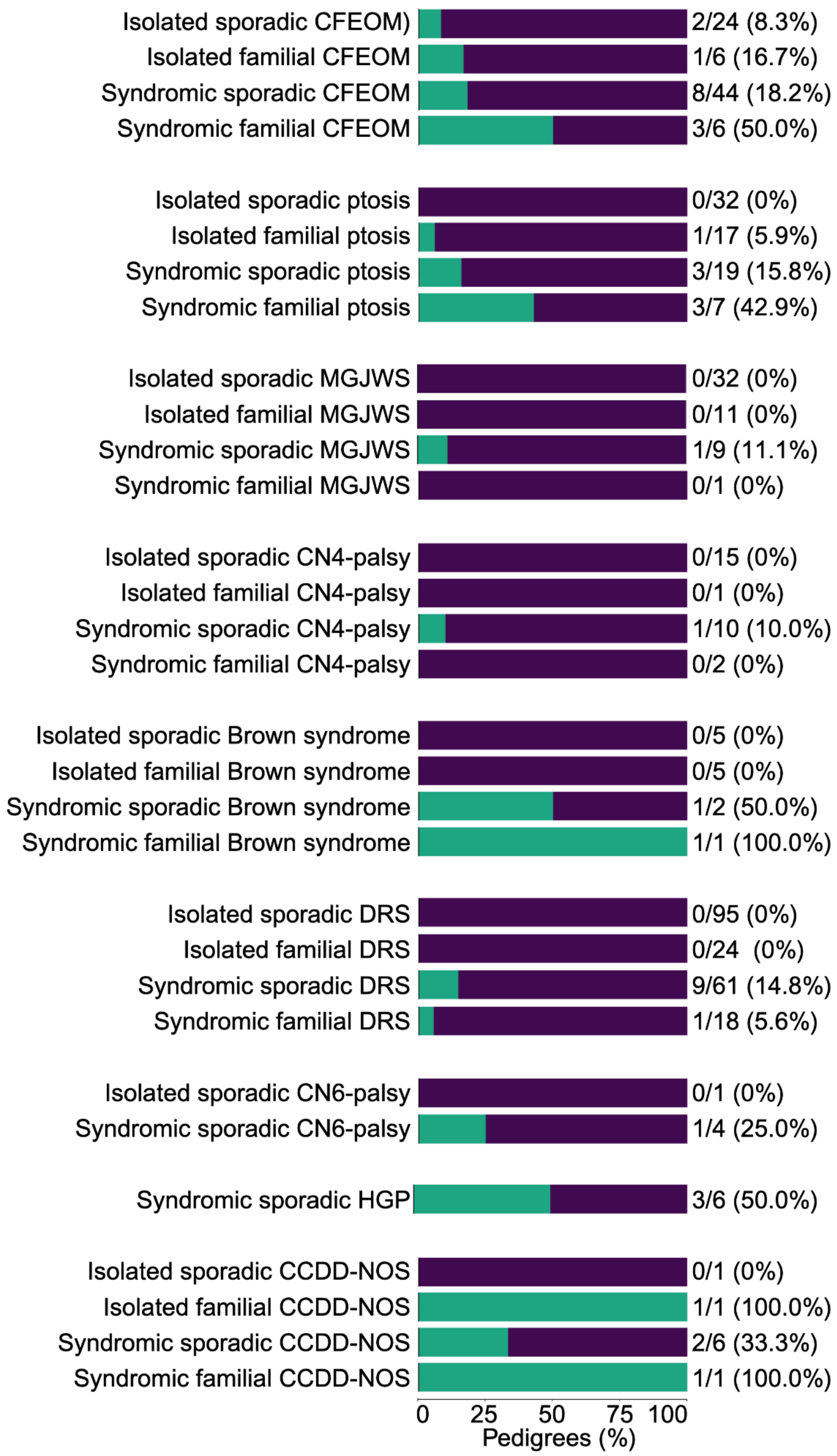

Key: green-counts of probands with ACMG/AMP/ClinGen-P/LP variants, purple-counts of probands without ACMG/AMP/ClinGen-P/LP variants. Abbreviations: ACMG=American College of Medical Genetics and Genomics, AMP=Association for Molecular Pathology, CCDD=congenital cranial dysinnervation disorder, CCDD-NOS=CCDD not otherwise specified, CFEOM=congenital fibrosis of the extraocular muscles, ClinGen=Clinical Genome Resource, CN4-palsy=fourth nerve palsy, CN6-palsy=sixth nerve palsy, DRS=Duane retraction syndrome, HGP=horizontal gaze palsy, LP=likely pathogenic ACMG/AMP/ClinGen classification, MGJWS=Marcus Gunn jaw-winking synkinesis, oCCDD=ocular congenital cranial dysinnervation disorder, P=pathogenic ACMG/AMP/ClinGen classification, Ptosis=congenital ptosis.

medRxiv preprint doi: https://doi.org/10.1101/2024.03.22.24304594; this version posted March 26, 2024. The copyright holder for this preprint (which was not certified by peer review) is the author/funder, who has granted medRxiv a license to display the preprint in perpetuity.
It is made available under a CC-BY 4.0 International license .

SUPPLEMENTARY TABLES

Supplementary Table 2. Sequencing, demographics, and oCCDD information among 467 probands

	CFEOM (n=80)	Ptosis (n=75)	MGJWS (+) ptosis (n=40)	MGJWS (-) ptosis (n=11)	INV-MGJWS (-) ptosis (n=2)	CN4-palsy (n=28)	Brown syndrome (n=13)	DRS (n=198)	CN6- palsy (n=5)	HGP (n=6)	CCDD-NOS (n=9)	TOTAL (n=467)
**Sequencing type**												
ES	26 (32.5%)	58 (77.3%)	19 (47.5%)	4 (36.4%)	2 (100%)	12 (42.9%)	8 (61.5%)	95 (48%)	4 (80%)	4 (66.7%)	8 (88.9%)	240 (51.4%)
GS	54 (67.5%)	17 (22.7%)	21 (52.5%)	7 (63.6%)	0 (0%)	16 (57.1%)	5 (38.5%)	103 (52%)	1 (20%)	2 (33.3%)	1 (11.1%)	227 (48.6%)
**Sequencing structure**												
Singleton	30 (37.5%)	38 (50.7%)	13 (32.5%)	4 (36.4%)	1 (50%)	4 (14.3%)	2 (15.4%)	76 (38.4%)	3 (60%)	3 (50%)	5 (55.6%)	179 (38.3%)
Duo	2 (2.5%)	2 (2.7%)	2 (5%)	0 (0%)	0 (0%)	0 (0%)	2 (15.4%)	4 (2%)	0 (0%)	0 (0%)	0 (0%)	12 (2.6%)
Trio	41 (51.3%)	28 (37.3%)	21 (52.5%)	4 (36.4%)	1 (50%)	22 (78.5%)	6 (46.1%)	100 (50.5%)	2 (40%)	3 (50%)	4 (44.4%)	232 (49.7%)
Quad	5 (6.2%)	4 (5.3%)	3 (7.5%)	2 (18.1%)	0 (0%)	1 (3.6%)	2 (15.4%)	6 (3%)	0 (0%)	0 (0%)	0 (0%)	23 (4.9%)
Other (>4)	2 (2.5%)	3 (4%)	1 (2.5%)	1 (9.1%)	0 (0%)	1 (3.6%)	1 (7.7%)	12 (6.1%)	0 (0%)	0 (0%)	0 (0%)	21 (4.5%)
**Sporadic vs. familial oCCDD breakdown**												
Sporadic	68 (85%)	51 (68%)	31 (77.5%)	8 (72.7%)	2 (100%)	25 (89.3%)	7 (53.9%)	156 (78.8%)	5 (100%)	6 (100%)	7 (77.8%)	366 (78.4%)
Familial	12 (15%)	24 (32%)	9 (22.5%)	3 (27.3%)	0 (0%)	3 (10.7%)	6 (46.1%)	42 (21.2%)	0 (0%)	0 (0%)	2 (22.2%)	101 (21.6%)
**Syndromic vs. isolated oCCDD breakdown**												
Syndromic	50 (62.5%)	26 (34.7%)	8 (20%)	2 (18.2%)	0 (0%)	12 (42.9%)	3 (23.1%)	79 (39.9%)	4 (80%)	6 (100%)	7 (77.8%)	197 (42.2%)
Isolated	30 (37.5%)	49 (65.3%)	32 (80%)	9 (81.8%)	2 (100%)	16 (57.1%)	10 (76.9%)	119 (60.1%)	1 (20%)	0 (0%)	2 (22.2%)	270 (57.8%)
**Combined sporadic/ familial and isolated/ syndromic oCCDD breakdown**												
Isolated Familial	6 (7.5%)	17 (22.7%)	8 (20%)	3 (27.3%)	0 (0%)	1 (3.6%)	5 (38.5%)	24 (12.1%)	0 (0%)	0 (0%)	1 (11.1%)	65 (13.9%)
Isolated Sporadic	24 (30%)	32 (42.7%)	24 (60%)	6 (54.5%)	2 (100%)	15 (53.6%)	5 (38.5%)	95 (48%)	1 (20%)	0 (0%)	1 (11.1%)	205 (43.9%)
Syndromic Familial	6 (7.5%)	7 (9.3%)	1 (2.5%)	0 (0%)	0 (0%)	2 (7.1%)	1 (7.6%)	18 (9.1%)	0 (0%)	0 (0%)	1 (11.1%)	36 (7.7%)
Syndromic Sporadic	44 (55%)	19 (25.3%)	7 (17.5%)	2 (18.2%)	0 (0%)	10 (35.7%)	2 (15.4%)	61 (30.8%)	4 (80%)	6 (100%)	6 (66.7%)	161 (34.5%)
**oCCDD laterality**												
Unilateral	31 (38.8%)	55 (73.3%)	30 (75%)	8 (72.7%)	1 (50%)	19 (67.9%)	9 (69.2%)	124 (62.6%)	2 (40%)	0 (0%)	4 (44.4%)	283 (60.6%)
Bilateral	46 (57.5%)	16 (21.3%)	1 (2.5%)	0 (0%)	0 (0%)	3 (10.7%)	2 (15.4%)	47 (23.7%)	3 (60%)	4 (66.7%)	4 (44.4%)	126 (27%)
Laterality ND	3 (3.7%)	4 (5.4%)	9 (22.5%)	3 (27.3%)	1 (50%)	6 (21.4%)	2 (15.4%)	27 (13.7%)	0 (0%)	2 (33.3%)	1 (11.2%)	58 (12.4%)
**Documented synkinesis**												
Synkinesis	23 (28.8%)	1 (1.3%)	40 (100%)	11 (100%)	2 (100%)	0 (0%)	1 (7.7%)	198 (100%)	0 (0%)	0 (0%)	8 (88.9%)	284 (60.8%)
No Synkinesis	57 (71.2%)	74 (98.7%)	0 (0%)	0 (0%)	0 (0%)	28 (100%)	12 (92.3%)	0 (0%)	5 (100%)	6 (100%)	1 (11.1%)	183 (39.2%)

Abbreviations: CCDD=congenital cranial dysinnervation disorder, CCDD-NOS=CCDD not otherwise specified, CFEOM=congenital fibrosis of the extraocular muscles, CN4-palsy=fourth nerve palsy, CN6-palsy=sixth nerve palsy, DRS=Duane retraction syndrome, ES=exome sequencing, GS=genome sequencing, HGP=horizontal gaze palsy, INV-MGJWS(-)ptosis=inverse Marcus Gunn jaw-winking synkinesis without congenital ptosis, MGJWS(+)ptosis=Marcus Gunn jaw-winking synkinesis with congenital ptosis, MGJWS(-)ptosis=Marcus Gunn jaw-winking synkinesis without congenital ptosis, ND=not described, oCCDD=ocular congenital cranial dysinnervation disorder, ptosis=congenital ptosis. Results are provided as numbers and percentages of individuals with each feature in each oCCDD diagnostic category.

medRxiv preprint doi: https://doi.org/10.1101/2024.03.22.24304594; this version posted March 26, 2024. The copyright holder for this preprint (which was not certified by peer review) is the author/funder, who has granted medRxiv a license to display the preprint in perpetuity.
It is made available under a CC-BY 4.0 International license .

Supplementary Table 3. Syndromic features among the 197 probands with syndromic oCCDDs

Diagnosis	CFEOM (n=50)	Ptosis (n=26)	MGJWS (+)ptosis (n=8)	MGJWS (-)ptosis (n=2)	CN4-palsy (n=12)	Brown syndrome (n=3)	DRS (n=79)	CN6-palsy (n=4)	HGP (n=6)	CCDD-NOS (n=7)	TOTAL (n=197)
Facial paralysis	6 (12.0%)	1 (3.8%)	0 (0%)	0 (0%)	1 (8.3%)	0 (0%)	5 (6.3%)	0 (0%)	1 (16.7%)	2 (28.6%)	16 (8.1%)
Hearing impairment	7 (14.0%)	3 (11.5%)	0 (0%)	0 (0%)	1 (8.3%)	1 (33.3%)	12 (15.2%)	2 (50.0%)	0 (0%)	0 (0%)	26 (13.2%)
Lower CN (IX-XII)	4 (8.0%)	2 (7.7%)	0 (0%)	0 (0%)	0 (0%)	0 (0%)	1 (1.3%)	2 (50.0%)	1 (16.7%)	0 (0%)	10 (5.1%)
CNS structural/ functional malf	40 (80.0%)	14 (53.8%)	3 (37.5%)	1 (50%)	6 (50.0%)	1 (33.3%)	33 (41.8%)	3 (75.0%)	3 (50.0%)	5 (71.4%)	109 (55.3%)
Additional PNS/ muscle/ connective tissue	18 (36.0%)	6 (23.1%)	2 (25.0%)	0 (0%)	2 (16.7%)	2 (66.7%)	31 (39.2%)	2 (50.0%)	4 (66.7%)	6 (85.7%)	73 (37.1%)
Craniofacial	20 (40.0%)	9 (34.6%)	2 (25.0%)	1 (50%)	5 (41.7%)	1 (33.3%)	27 (34.2%)	1 (25.0%)	2 (33.3%)	5 (71.4%)	73 (37.1%)
Dysmorph-other	9 (18.0%)	3 (11.5%)	1 (12.5%)	0 (0%)	3 (25.0%)	1 (33.3%)	19 (24.1%)	1 (25.0%)	1 (16.7%)	0 (0%)	38 (19.3%)
Skeletal	8 (16.0%)	2 (7.7%)	0 (0%)	0 (0%)	1 (8.3%)	1 (33.3%)	31 (39.2%)	0 (0%)	2 (33.3%)	3 (42.9%)	48 (24.4%)
Scoliosis	4 (8.0%)	1 (3.8%)	0 (0%)	0 (0%)	1 (8.3%)	0 (0%)	10 (12.7%)	0 (0%)	4 (66.7%)	4 (57.1%)	24 (12.2%)
Pulmonary/ Lung/ Respiratory	7 (14.0%)	4 (15.4%)	1 (12.5%)	1 (50%)	1 (8.3%)	2 (66.7%)	4 (5.1%)	0 (0%)	0 (0%)	3 (42.9%)	23 (11.7%)
Cardio	9 (18.0%)	8 (30.8%)	1 (12.5%)	2 (100%)	1 (8.3%)	1 (33.3%)	18 (22.8%)	0 (0%)	1 (16.7%)	3 (42.9%)	44 (22.3%)
GI	11 (22.0%)	7 (26.9%)	4 (50.0%)	1 (50%)	0 (0%)	1 (33.3%)	17 (21.5%)	0 (0%)	0 (0%)	4 (57.1%)	45 (22.8%)
Renal/ urinary/ genital	9 (18.0%)	5 (19.2%)	2 (25.0%)	0 (0%)	1 (8.3%)	0 (0%)	15 (19.0%)	0 (0%)	0 (0%)	1 (14.3%)	33 (16.8%)
Endocrine	6 (12.0%)	1 (3.8%)	1 (12.5%)	1 (50%)	0 (0%)	0 (0%)	14 (17.7%)	1 (25.0%)	0 (0%)	2 (28.6%)	26 (13.2%)
Skin/ hair/ teeth/ nails	10 (20.0%)	7 (26.9%)	0 (0%)	0 (0%)	1 (8.3%)	0 (0%)	22 (27.8%)	0 (0%)	2 (33.3%)	2 (28.6%)	44 (22.3%)
Other	0 (0%)	1 (3.8%)	0 (0%)	0 (0%)	0 (0%)	0 (0%)	4 (5.1%	0 (0%)	0 (0%)	0 (0%)	5 (2.5%)

Abbreviations: Cardio=cardiovascular, CCDD=congenital cranial dysinnervation disorder, CCDD-NOS= CCDD not otherwise specified, CFEOM=congenital fibrosis of the extraocular muscles, CN=cranial nerve, CNS=central nervous system, CN4-palsy=fourth nerve palsy, CN6-palsy=sixth nerve palsy, DRS=Duane retraction syndrome, Dysmorph=dysmorphology, GI=gastrointestinal, HGP=horizontal gaze palsy, Malf=malformation, MGJWS(+)ptosis=Marcus Gunn jaw-winking synkinesis with congenital ptosis, MGJWS(-)ptosis=Marcus Gunn jaw-winking synkinesis without congenital ptosis, oCCDD=ocular congenital cranial dysinnervation disorder, PNS=peripheral nervous system, ptosis=congenital ptosis. Note: all inverse MGJWS(-)ptosis cases were nonsyndromic and thus were not included in this table. Results are provided as numbers and percentages of individuals with each feature in each oCCDD diagnostic category.

medRxiv preprint doi: https://doi.org/10.1101/2024.03.22.24304594; this version posted March 26, 2024. The copyright holder for this preprint (which was not certified by peer review) is the author/funder, who has granted medRxiv a license to display the preprint in perpetuity.
It is made available under a CC-BY 4.0 International license .

Supplementary Table 4. CODA analysis of co-occurring syndromic phenotypes

oCCDD Diagnosis	Defect A	Defect B	Defect C	Defect D	Defect E	Number of probands with phenotype combination	OEun	OEadj
DRS	4	5	6	7	8	4	9.58	5.30
DRS	4	5	6	7	9	4	29.69	11.39
DRS	4	5	6	7	15	3	10.12	5.86
DRS	4	5	6	8	9	3	13.65	6.11
DRS	4	5	6	8	10	3	34.12	15.08
DRS	4	5	6	9	12	3	24.89	8.75
DRS	4	5	8	9	12	3	21.67	12.02
DRS	5	6	7	8	15	3	10.77	6.76
DRS	5	6	7	12	15	3	19.65	10.72
DRS	6	7	8	12	15	3	19.65	8.94
DRS	6	7	9	12	15	3	60.90	17.42
DRS	4	6	7	8	14	3	15.90	8.83
DRS	1	4	5	15		3	13.14	6.08
DRS	4	5	6	12		4	4.20	3.13
DRS	4	5	6	15		5	4.06	3.02
DRS	4	5	8	15		3	2.12	1.85
DRS	4	5	9	12		4	11.34	7.77
DRS	4	5	10	12		3	21.26	12.57
DRS	4	5	12	15		3	3.87	2.47
DRS	4	6	7	8		6	5.64	5.15
DRS	4	6	7	12		3	5.14	3.53
DRS	4	6	8	12		3	3.15	2.68
DRS	5	6	8	12		3	3.35	2.92
DRS	5	7	8	15		4	4.91	5.14
DRS	6	7	8	9		3	9.30	4.05
DRS	6	7	8	12		4	7.29	4.76
DRS	6	8	9	12		3	10.40	6.01
DRS	7	8	9	12		3	14.77	6.42
DRS	4	5	6	13		3	3.57	2.46
DRS	4	5	9	13		3	9.64	6.04
DRS	4	5	13	15		3	4.38	2.96
DRS	5	6	12	13		3	6.93	6.59
DRS	5	6	8	13		4	5.07	4.83
DRS	6	7	8	11		3	5.17	3.00
DRS	6	8	11	13		4	8.73	6.72

medRxiv preprint doi: https://doi.org/10.1101/2024.03.22.24304594; this version posted March 26, 2024. The copyright holder for this preprint (which was not certified by peer review) is the author/funder, who has granted medRxiv a license to display the preprint in perpetuity.
It is made available under a CC-BY 4.0 International license .

DRS	2	4	5	6		4	5.95	4.45
DRS	2	4	6	8		3	4.46	3.94
DRS	4	6	7	11		3	4.85	3.78
DRS	4	6	8	11		3	2.98	2.30
DRS	4	8	11	14		3	5.74	8.98
DRS	4	5	6	14		3	3.83	2.53
DRS	2	4	11			3	2.63	3.34
DRS	2	4	12			3	2.78	2.92
DRS	4	11	12			4	2.47	2.86
DRS	1	4	5			4	4.88	4.15
DRS	4	12	15			4	2.02	2.13
DRS	5	7	9			5	5.30	4.87
DRS	5	7	12			4	2.49	2.52
DRS	5	8	9			4	2.60	3.06
DRS	5	9	15			3	2.75	2.11
DRS	7	9	12			4	7.73	5.57
DRS	6	7	13			3	2.43	2.18
DRS	4	5	11			4	1.36	1.38
DRS	5	6	11			4	1.66	1.79
DRS	5	13	15			4	2.44	2.53
DRS	6	13	15			3	2.10	2.21
DRS	11	12	14			3	4.37	4.00
DRS	5	8	11			3	1.08	1.42
DRS	5	11	12			3	1.97	2.17
DRS	5	11	15			3	1.53	1.66
DRS	8	11	12			3	1.97	2.17
DRS	4	5	14			5	2.18	2.30
DRS	4	12	14			4	3.18	3.17
DRS	8	12	14			3	2.54	2.96
DRS	4	14	15			3	1.84	2.05
DRS	2	6	14			3	4.13	4.55
DRS	4	15				13	1.41	2.16
DRS	9	15				4	1.44	1.84
DRS	2	13				3	1.32	1.78
DRS	11	14				5	1.57	1.93
DRS	7	11				5	1.15	1.42
DRS	8	11				8	1.13	1.59
DRS	8	13				7	1.19	1.71
DRS	11	13				5	1.46	1.79

medRxiv preprint doi: https://doi.org/10.1101/2024.03.22.24304594; this version posted March 26, 2024. The copyright holder for this preprint (which was not certified by peer review) is the author/funder, who has granted medRxiv a license to display the preprint in perpetuity.
It is made available under a CC-BY 4.0 International license .

DRS	6	14				6	1.25	1.61
DRS	8	14				5	0.91	1.24
CFEOM	4	5	6	7	8	4	24.11	12.22
CFEOM	4	5	6	7	15	3	14.47	12.81
CFEOM	4	5	6	8	15	3	16.28	7.62
CFEOM	4	5	7	8	15	3	36.17	15.63
CFEOM	4	6	7	8	15	3	32.55	12.33
CFEOM	5	6	7	8	15	3	72.34	16.56
CFEOM	3	4	5	6	10	3	46.50	19.63
CFEOM	3	4	5	6	14	3	54.25	16.42
CFEOM	3	4	5	10	14	3	155.01	32.00
CFEOM	3	4	6	10	14	3	139.51	31.73
CFEOM	3	5	6	10	14	3	310.02	33.25
CFEOM	4	5	6	8	14	3	27.13	9.05
CFEOM	4	5	6	10	14	3	31.00	16.50
CFEOM	3	4	5	6	12	3	29.59	17.68
CFEOM	4	5	6	13		3	2.89	3.30
CFEOM	4	6	7	13		3	5.79	4.95
CFEOM	4	6	8	13		3	6.51	4.67
CFEOM	4	6	8	15		4	7.81	7.50
CFEOM	2	4	5	6		3	3.72	3.73
CFEOM	2	4	6	10		3	9.57	6.76
CFEOM	2	4	6	14		3	11.16	7.21
CFEOM	3	4	5	6		4	8.68	9.51
CFEOM	4	5	6	14		4	5.79	6.60
CFEOM	4	5	8	10		3	9.30	6.53
CFEOM	4	5	6	11		3	2.89	3.02
CFEOM	4	5	8	11		3	7.23	5.28
CFEOM	4	5	11	12		3	5.26	6.06
CFEOM	4	6	7	11		3	5.79	5.57
CFEOM	4	6	10	12		4	8.12	8.82
CFEOM	4	6	11	12		3	4.73	4.62
CFEOM	4	6	11	14		3	8.68	7.56
CFEOM	4	6	11	13		3	5.79	5.57
CFEOM	4	6	13	14		3	8.68	6.61
CFEOM	4	6	12	13		3	4.73	4.62
CFEOM	4	6	13			6	2.08	3.49
CFEOM	4	6	15			5	1.56	2.43
CFEOM	5	6	13			4	3.09	3.33

medRxiv preprint doi: https://doi.org/10.1101/2024.03.22.24304594; this version posted March 26, 2024. The copyright holder for this preprint (which was not certified by peer review) is the author/funder, who has granted medRxiv a license to display the preprint in perpetuity.
It is made available under a CC-BY 4.0 International license .

CFEOM	1	4	5			3	1.74	2.32
CFEOM	1	4	6			3	1.56	2.13
CFEOM	2	4	6			6	2.68	4.08
CFEOM	3	4	5			4	3.47	5.09
CFEOM	3	4	6			4	3.13	4.33
CFEOM	4	5	10			4	1.98	2.79
CFEOM	4	6	10			5	2.23	3.20
CFEOM	4	6	14			5	2.60	4.03
CFEOM	4	5	11			5	1.93	2.99
CFEOM	4	5	12			5	1.58	2.26
CFEOM	4	6	11			5	1.74	2.58
CFEOM	4	6	12			6	1.70	2.49
CFEOM	4	7	12			3	1.89	2.27
CFEOM	4	9	12			3	4.26	4.99
CFEOM	4	10	11			4	3.97	5.07
CFEOM	4	12	15			4	2.27	2.88
CFEOM	2	4	7			3	2.98	3.80
CFEOM	1	4	12			3	2.84	3.26
CFEOM	2	4	12			3	2.44	2.59
CFEOM	1	5				4	1.85	2.34
CFEOM	4	9				4	1.25	1.90
Congenital ptosis	2	4	5	15		2	19.93	18.57
Congenital ptosis	4	5	10	13		2	20.92	17.89
Congenital ptosis	4	10	11	12		2	11.21	15.32
Congenital ptosis	2	4	6	12		2	13.28	11.42
Congenital ptosis	3	4	5	12		2	29.89	22.90
Congenital ptosis	4	6	11			2	1.34	1.78
Congenital ptosis	2	4	13			2	6.44	6.29
Congenital ptosis	4	5	13			3	4.83	5.70
Congenital ptosis	4	6	12			3	2.30	3.63
Congenital ptosis	4	11	12			3	2.59	3.78
Congenital ptosis	4	12	13			2	2.76	3.08
Congenital ptosis	4	6	15			2	1.53	2.42

medRxiv preprint doi: https://doi.org/10.1101/2024.03.22.24304594; this version posted March 26, 2024. The copyright holder for this preprint (which was not certified by peer review) is the author/funder, who has granted medRxiv a license to display the preprint in perpetuity.
It is made available under a CC-BY 4.0 International license .

Congenital ptosis	6	7	15			2	7.15	10.18
Congenital ptosis	4	5	11			2	2.01	2.36
Congenital ptosis	6	11				3	1.08	1.67
Congenital ptosis	2	4				3	1.86	2.87
Congenital ptosis	4	10				3	1.39	2.09
Congenital ptosis	4	15				3	0.80	1.19
Congenital ptosis	6	8				2	2.89	3.97
Congenital ptosis	6	15				3	1.24	1.80
Congenital ptosis	7	15				3	3.71	6.19
Congenital ptosis	12	15				2	1.06	1.49

Abbreviations: CCDD-congenital cranial dysinnervation disorder, CFEOM-congenital fibrosis of the extraocular muscles, DRS-Duane retraction syndrome, oCCDD-ocular congenital cranial dysinnervation disorder, OEadj-adjusted observed/expected ratio, OEun-unadjusted observed/expected ratio, 1-facial paralysis, 2-hearing impairment, 3-lower cranial nerve, 4-central nervous system structural/functional malformation, 5-peripheral nervous system/muscle/connective tissue, 6-craniofacial, 7-non-craniofacial dysmorphisms, 8- skeletal (non-scoliosis), 9-skeletal (scoliosis), 10-pulmonary/ lung/ respiratory, 11-cardiovascular, 12-gastrointestinal (GI), 13- renal/urinary/genital, 14-endocrine, 15-skin/ hair/ teeth/nails.

Supplementary Table 11. Additional novel oCCDD candidate genes/variants of uncertain significance that may merit additional study

Gene	Variant	ACMG/AMP Classification	Category	Pedigrees	Diagnosis
KIF21A	NP_001166935.1:p.(Pro839Leu)	VUS	[oCCDD+, Syndrome+/-]	ENG_CHF	Syndromic sporadic CFEOM
ROBO3	NC_000011.10(NM_022370.4): c.2804-7_2804-6inv	VUS	[oCCDD+, Syndrome+/-]	193	Syndromic sporadic HGP
MPZ	NP_000521.2:p.(Ile62Met)	VUS	[oCCDD(+), Syndrome+]	ENG_IV	Isolated sporadic MGJWS(+)ptosis
MPZ	NP_000521.2:p.(Leu190Pro)	VUS	[oCCDD(+), Syndrome+]	14	Isolated sporadic MGJWS(+)ptosis
MYH10	NP_001242941.1:p.(Asp74Asn)	VUS	[oCCDD(+), Syndrome+]	ENG_CGO	Isolated sporadic MGJWS(-)ptosis
MYH10	NP_001242941.1:p.(Gly313Val)	VUS	[oCCDD(+), Syndrome+]	ENG_CKM	Isolated familial CFEOM
MYH10	NP_001242941.1:p.(Asn322Ser)	VUS	[oCCDD(+), Syndrome+]	ENG_ASW	Syndromic sporadic CFEOM
MYH10	NP_001242941.1:p.(Arg1735Gln)	VUS	[oCCDD(+), Syndrome+]	ENG_PJ	Isolated sporadic MGJWS(+)ptosis
MYH10	NP_001242941.1:p.(Arg1975His)	VUS	[oCCDD(+), Syndrome+]	ENG_YY	Isolated familial MGJWS(+)ptosis
TGFBR2	NP_003233.4:p.(Arg356Trp)	VUS	[oCCDD(+), Syndrome+]	ENG_1788	Isolated sporadic DRS
TUBB4A	NP_006078.2:p.(Met267Thr)	VUS	[oCCDD(+), Syndrome+]	216	Isolated familial Brown syndrome
ZNF462	NP_067047.4:p.(Thr976Ala)	VUS	[oCCDD(+), Syndrome+]	ENG_0308	Isolated sporadic congenital ptosis

medRxiv preprint doi: https://doi.org/10.1101/2024.03.22.24304594; this version posted March 26, 2024. The copyright holder for this preprint (which was not certified by peer review) is the author/funder, who has granted medRxiv a license to display the preprint in perpetuity.
It is made available under a CC-BY 4.0 International license .

ZNF462	NP_067047.4:p.(Arg1924His)	VUS	[oCCDD(+), Syndrome+]	ENG_0243	Syndromic sporadic congenital ptosis
GNAS	NP_057676.1:p.(Glu102Gln)	VUS	[oCCDD-, Syndrome+]	ENG_KS	Syndromic sporadic DRS
GNAS	NP_057676.1:p.(Gly238Glu)	VUS	[oCCDD-, Syndrome+]	ENG_UE	Isolated sporadic DRS
GNAS	NP_536350.2:p.(Pro531Ser)	VUS	[oCCDD-, Syndrome+]	ENG_JU	Isolated sporadic DRS
GNAS	NP_536350.2:p.(Asp573His)	VUS	[oCCDD-, Syndrome+]	ENG_AAJ	Isolated sporadic DRS
SLC12A5	NP_065759.1:p.(Gly647Ala)	VUS	[oCCDD-, Syndrome+]	260	Syndromic familial CFEOM
ACTR1B	NP_005726.1:p.(Phe211Leu)	VUS	[oCCDD-, Syndrome-]	ENG_BAE	Isolated sporadic DRS
ACTR1B	NP_005726.1:p.(Arg336Gly)	VUS	[oCCDD-, Syndrome-]	ENG_CMJ	Isolated sporadic DRS
CTNNA1	NP_001894.2:p.(Asp813Ala)	VUS	[oCCDD-, Syndrome-]	99	Syndromic sporadic CFEOM
CUX1	NP_853530.2:p.(Met231Val)	VUS	[oCCDD-, Syndrome-]	ENG_PQ	Isolated sporadic DRS
CUX1	NP_853530.2:p.(Ile1274Val)	VUS	[oCCDD-, Syndrome-]	ENG_GH	Isolated sporadic DRS
CUX1	NP_853530.2:p.(Glu1254Lys)	VUS	[oCCDD-, Syndrome-]	230	Isolated familial DRS
FER	NP_005237.2:p.(Thr628Ile)	VUS	[oCCDD-, Syndrome-]	ENG_1616	Isolated sporadic DRS
FER	NP_005237.2:p.(Gln629His)	VUS	[oCCDD-, Syndrome-]	ENG_1637	Isolated sporadic DRS
FGF21	NP_061986.1:p.(Arg45Trp)	VUS	[oCCDD-, Syndrome-]	91	Syndromic sporadic CFEOM
KIF5C	NP_004513.1:p.(Glu483Gly)	VUS	[oCCDD-, Syndrome-]	ENG_UV	Isolated sporadic CCDD-NOS
KIF5C	NP_004513.1:p.(Thr872Met)	VUS	[oCCDD-, Syndrome-]	ENG_ABE	Syndromic familial DRS
KIF5C	NP_004513.1:p.(Arg894Cys)	VUS	[oCCDD-, Syndrome-]	ENG_1561	Syndromic sporadic DRS
KLB	NP_783864.1:p.(Arg490Gln)	VUS	[oCCDD-, Syndrome-]	ENG_CKP	Syndromic sporadic CCDD-NOS
NES	NP_006608.1:p.(Glu8Val)	VUS	[oCCDD-, Syndrome-]	251	Isolated familial CFEOM
OLIG2	NP_005797.1:p.(Arg156Leu)	VUS	[oCCDD-, Syndrome-]	ENG_ET	Isolated familial DRS
SEMA3F	NP_004177.3:p.(Ser630Ter)	VUS	[oCCDD-, Syndrome-]	ENG_CMK	Syndromic sporadic CFEOM
TUBB	NP_821133.1:p.(Gly400Arg)	VUS	[oCCDD-, Syndrome-]	ENG_0678	Isolated sporadic CFEOM

Abbreviations: ACMG=American College of Medical Genetics and Genomics, AMP=Association for Molecular Pathology, CCDD-NOS=CCDD not otherwise specified, CFEOM=congenital fibrosis of the extraocular muscles, DRS=Duane retraction syndrome, HGP=horizontal gaze palsy, MGJWS(+)ptosis=Marcus Gunn jaw-winking synkinesis with congenital ptosis, MGJWS(-)ptosis=Marcus Gunn jaw-winking synkinesis without congenital ptosis, oCCDD=ocular congenital cranial dysinnervation disorder, VUS=variant of uncertain significance. Genes are delineated into five categories, defined in the main text and in Figure 3.
